# M2 macrophage-based classification identifies DOK3 as a driver of pro-tumoral polarization and migration in glioblastoma

**DOI:** 10.3389/fimmu.2026.1725581

**Published:** 2026-01-29

**Authors:** Chang-Yuan Ren, Ji Shi, Chang-Lin Yang, Jin-Hao Zhang, Wen-Lu Tan, Han-Xiao Zhou, Pan-Yu Ren, Josip Cvitković, Wen-Hua Fan, Ying Zhang, Zheng Zhao

**Affiliations:** 1 Beijing Neurosurgical Institute, Capital Medical University, Beijing, China; 2 Beijing Tiantan Hospital, Capital Medical University, Beijing, China; 3 Department of Neurosurgery, Liaoning Cancer Hospital & Institute/Cancer Hospital of Dalian University of Technology/Cancer Hospital of China Medical University, Shenyang, China; 4 Chinese Glioma Genome Atlas (CGGA) Network, Beijing, China; 5 Asian Glioma Genome Atlas (AGGA) Network, Beijing, China; 6 Chinese Neuro-Oncology Genome Atlas (CGGA-CNS) Network, Beijing, China

**Keywords:** DOK3, glioblastoma, M2 macrophage, pro-tumoral polarization, tumor microenvironment

## Abstract

**Background:**

Glioblastoma (GBM) is the most aggressive primary brain tumor, characterized by limited therapeutic options and dismal prognosis. Among the components of the immunosuppressive tumor microenvironment (TME), M2-polarized macrophages are pivotal mediators of tumor progression, yet the molecular mechanisms underlying their polarization and pro-tumoral functions remain inadequately understood.

**Methods:**

We integrated bulk and single-cell RNA sequencing datasets from CGGA, TCGA, and GSE131928. M2 macrophage infiltration was quantified using the xCell algorithm, and unsupervised clustering of M2-associated genes was first performed to define macrophage-centered immune subtypes, further refined by XGBoost and LASSO modeling. Based on the molecular features of the most immunosuppressive subtype, a macrophage-associated risk score based on CTSB, LITAF, and DOK3 was constructed and validated across independent cohorts for prognostic and immune relevance. Functional validation was performed by silencing DOK3 in THP-1–derived macrophages, followed by co-culture with glioma cells to assess macrophage polarization and tumor cell behavior.

**Results:**

Elevated M2 macrophage infiltration correlated with reduced tumor purity, spatial heterogeneity, and worse survival. Three immune subtypes (C1–C3) were identified; notably, the C1 subtype exhibited the highest M2 infiltration, strongest immunosuppressive features, and poorest prognosis. The derived macrophage-based risk score robustly stratified patient survival and correlated with CD163 expression and immune checkpoint activation. Single-cell analysis revealed predominant DOK3 expression in macrophages and microglia. Functional assays demonstrated that DOK3 knockdown reduced CD163 expression and attenuated glioma cell invasiveness, supporting its role in promoting M2 polarization and tumor aggressiveness.

**Conclusion:**

This integrative analysis identifies DOK3 as a pivotal regulator of M2 macrophage polarization and a driver of glioblastoma progression. Together, immune subtyping and the simplified macrophage-based risk model represent complementary strategies, with the latter providing a practical tool for prognostic stratification. Targeting DOK3 offers a promising therapeutic strategy to reprogram the TME and improve clinical outcomes in patients with GBM.

## Introduction

Glioma is the most common and highly aggressive primary malignant brain tumor, accounting for approximately 80% of all primary malignant brain tumors (1, 2). According to the 2021 World Health Organization (WHO) classification of tumors of the central nervous system (CNS), adult-type diffuse gliomas are classified into three major categories: IDH-mutant oligodendroglioma, IDH-mutant astrocytoma, and IDH-wildtype glioblastoma (GBM) (5). Despite advances in multimodal treatment–including maximal surgical resection, radiotherapy, and temozolomide-based chemotherapy–the prognosis of GBM remains dismal, with a median overall survival (OS) of only 14–18 months and a 5-year survival rate below 10% (3). The highly infiltrative nature of GBM hampers completed surgical resection, contributing to inevitable recurrence and resistance to conventional therapies (4).

Recent molecular and immunological profiling has revealed that GBM harbors a profoundly immunosuppressive tumor microenvironment (TME), enriched with regulatory immune cells and inhibitory soluble factors that dampen the activity of cytotoxic T cells and natural killer (NK) cells, thereby promoting immune evasion and disease progression (6–8). This immunosuppressive milieu not only accelerates tumor growth but also limits the efficacy of emerging immunotherapeutic strategies, highlighting the urgent need for precise prognostic stratification systems and robust molecular target to guide personalized treatment strategies.

Tumor-associated macrophages (TAMs) are the dominant immune cell component within the GBM microenvironment, with the M2-polarized phenotype constituting the predominant and functionally pro-tumoral subset (9). High densities of M2 macrophages have been strongly associated with higher WHO grades, enhanced invasiveness, and poorer clinical outcomes (10). Mechanistically, M2 macrophages exert potent immunosuppressive effects by secreting IL10, TGF-β, Arg-1, and VEGF, upregulating PD-L1 expression (11), promoting T-cell exhaustion, and expanding regulatory T-cells (Treg) populations (12). Enrichment of M2 macrophages in glioma tissues has been correlated with reduced IFN-γ expression, indicative of impaired T-cell activity *in vivo* (13). Furthermore, M2 macrophages suppress NK cells cytotoxicity through TGF-β–mediated downregulation of the activating receptor NKG2D (14, 15), a key mechanism of tumor immune escape (16). Notably, blocking TGF-β signaling in preclinical glioma models restores NKG2D expression and enhances NK cell-mediated tumor killing (17). These findings collectively highlight the central role of M2 macrophages as orchestrators of GBM immunosuppression and tumor progression, positioning them as compelling targets for therapeutic intervention. Preclinical studies using CSF1R inhibitors, for example, have demonstrated the potential to reprogram M2 macrophages toward a pro-inflammatory M1 phenotype, thereby enhancing antitumor immunity (16).

In this study, we combined integrative multi-omics analyses with functional experiments to investigate the molecular drivers of M2 macrophage polarization in GBM. We identified *DOK3* as a key regulator of pro-tumoral polarization and glioma cell migration, providing mechanistic insights into macrophage-mediated tumor progression and highlighting *DOK3* as a promising immunotherapeutic target for patients with IDH-wildtype GBM.

## Materials and methods

### Data acquisition

Multiple glioblastoma (GBM) datasets were obtained from publicly available, authoritative databases. RNA sequencing data and corresponding clinical information from 205 GBM patients were retrieved from The Cancer Genome Atlas (TCGA, http://cancergenome.nih.gov/) (18) and used as the training cohort for feature selection and model construction. An additional 74 GBM samples from the Chinese Glioma Genome Atlas (CGGA, http://cgga.org.cn/) (19, 20) served as an independent external validation cohort to evaluate the robustness and generalizability of the model. Clinical variables included, but were not limited to, age, sex, IDH mutation status, MGMT promoter methylation status, chemoradiotherapy, and survival outcomes such as overall survival (OS) and progression-free survival (PFS). Only IDH-wildtype GBM patients were enrolled in this study.

To investigate the spatial distribution of M2 macrophages in different histological regions, we analyzed data from the Ivy Glioblastoma Atlas Project (http://glioblastoma.alleninstitute.org/) (21). This dataset comprises gene expression profiles from five anatomically defined regions: (1) cellular tumor (CT), (2) infiltrating tumor (IT), (3) leading edge (LE), (4) microvascular proliferation (MP), and (5) pseudopalisading cells (PC).

To further exploring the immune microenvironment at single-cell resolution, we incorporated single-cell RNA sequencing data (scRNA-seq) data from both the CGGA database and the GSE131928 dataset in the Gene Expression Omnibus (GEO) (https://www.ncbi.nlm.nih.gov/geo/) (22). These datasets include multiple primary and recurrent GBM samples, enabling high resolution characterization of immune cell heterogeneity and dynamics within the tumor microenvironment.

All datasets were accessed in accordance with their respective usage policies and ethical guidelines. The study was conducted in compliance with the principles of the Declaration of Helsinki.

### Estimation of immune infiltrates

Immune cell infiltration was quantified using the TIMER2.0 platform (http://timer.cistrome.org/) (23) and xCell algorithm (24) based on bulk RNA-seq data from both the TCGA, CGGA, and IVY cohorts. TIMER2.0 integrates multiple state-of-the-art deconvolution algorithms, including TIMER, CIBERSORT, quanTIseq, and MCP-counter, enabling robust and comprehensive estimates of immune cell abundance across tumor samples. These estimates serve as the basis for subsequent immunological analyses and correlation studies. To identify genes associated with M2 macrophage activity, Pearson correlation analysis was performed between gene expression levels and the M2 macrophages score in each dataset, with genes showing strong positive correlation (r > 0.5) in both cohorts retained for downstream analyses.

### Estimation of immune score, stromal score, and tumor purity

Immune score, stromal score, and tumor purity were calculated using the ESTIMATE algorithm based on transcriptomic data. Immune and stromal scores represent the relative infiltration levels of immune and stromal cells, respectively, while tumor purity reflects the estimated proportion of malignant tumor cells within each sample. These parameters were included in downstream analyses to characterize the tumor microenvironment and its association with immune subtypes and clinical outcomes.

### Unsupervised clustering and subtype identification

Genes significantly correlated with M2 macrophage infiltration (*R* > 0.5 and *p* < 0.05) in both the TCGA and CGGA datasets were subjected to unsupervised clustering using the *ConsensusClusterPlus* R package. To ensure the stability and predictive utility of the identified subtypes, a random forest classifier was trained using the TCGA cohorts and subsequently applied to the CGGA cohort for external validation. The robustness and separability of the resulting clusters were further assessed using principal component analysis (PCA).

### Risk model construction

To identify key genes predictive of the immunosuppressive subtype, we applied Extreme Gradient Boosting (XGBoost) and least absolute shrinkage and selection operator (LASSO) regression to the TCGA cohort. Three candidate genes–CTSB, LITAF, and DOK3–were selected to construct a macrophage-associated risk score model. The prognostic value of this model was evaluated using Kaplan-Meier survival analysis and univariate and multivariate Cox regression. In addition, correlations between the risk score and CD163 expression, immune checkpoint markers, and immune infiltration patterns were systematically analyzed to elucidate the immunological and clinical relevance of the model.

### Single-cell transcriptomic analysis

Single-cell RNA sequencing (scRNA-seq) data were processed using the Seurat R package (version 5.0.0) (25). Low-quality cells and lowly expressed genes were filtered out based on standard quality control metrics, followed by normalization and batch-effect correlation. Dimensionality reduction and clustering were performed using the Uniform Manifold Approximation and Projection (UMAP) algorithm. The expression patterns of key marker genes (CD68, TMEM119, CD163, and DOK3) was analyzed across different immune and non-immune cell clusters to characterize their cellular distribution and functional relevance.

### Cell lines

LN229 and THP-1 cells were purchased from the American Type Culture Collection (ATCC). LN229 is a well-established human glioma cell line derived from a patient with IDH-wildtype glioblastoma. LN229 cells were cultured in high-glucose Dulbecco’s Modified Eagle Medium (DMEM) supplemented with 10% fetal bovine serum (FBS) and 1% penicillin–streptomycin, and maintained at 37 °C in a humidified incubator with 5% CO_2_. THP-1 cells were maintained in RPMI-1640 medium supplemented with 10% fetal bovine serum (FBS), 1% penicillin–streptomycin, and 0.1 mM β-mercaptoethanol, and cultured under the same conditions (37 °C, 5% CO_2_, humidified atmosphere).

### Macrophage polarization and glioma cell co-culture assays

To induce differentiation of THP-1 cells into M0 macrophages, cells were treated with 185 ng/mL phorbol 12-myristate 13-acetate (PMA, dissolved in DMSO) for 12 h, resulting in an adherent phenotype (26). For M2 polarization, adherent cells were co-incubated with 20 ng/mL IL-4 and 20 ng/mL IL-13 in the continued presence of PMA for 48 hours (27). For gene silencing experiments, siRNA negative control (siRNA-NC) or siRNA targeting DOK3 (siRNA-DOK3) was transfected into the respective groups, followed by 48 hours of incubation. All LN229 cells used in the experiments were in the logarithmic growth phase and were subjected to conditioned medium treatment or co-culture with polarized THP-1–derived macrophages to evaluate the effects of macrophage polarization on glioma cell migration and invasion. For Transwell co-culture assays, 1 × 10^5^ control or DOK3-silenced M2 macrophages were seeded in the upper chamber, while 2 × 10^5^ LN229 cells were placed in the lower chamber, followed by incubation at 37 °C for 48 h prior to downstream analyses.

### Western-blot assay

After polarization to the M2 phenotype, THP-1 cells were transfected with siRNA targeting DOK3. Total protein was extracted using RIPA buffer, and protein concentrations were measured by BCA assay. Equal amounts of protein were separated by SDS-PAGE and transferred onto PVDF membranes. Membranes were blocked with 5% skim milk for 1 hour at room temperature, followed by overnight incubation at 4 °C with primary antibodies: DOK3 (Immunoway, YT1397, 1:1000), CD163 (Abcam, ab156769, 1:1000), and GAPDH (Immunoway, YM3029, 1:20000). After washing, membranes were incubated with HRP-conjugated secondary antibodies for 1 hour at room temperature. Protein bands were visualized using enhanced chemiluminescence (ECL) and imaged with a Bio-Rad imaging system.

### Immunofluorescence

Cells were fixed with 4% paraformaldehyde, permeabilized with Triton X-100, and blocked with goat serum. Cells were incubated overnight with primary antibodies against DOK3 and CD163, followed by incubation with Alexa Fluor-conjugated secondary antibodies. Nuclei were counterstained with DAPI, and fluorescence imaging was performed using a confocal microscope.

### Transwell migration assay

Transwell assays were performed using 8-μm pore size polycarbonate membrane inserts. LN229 glioma cells (4 × 10^4^ cells in 200 μL serum-free medium) were seeded into the upper chamber, while M2-polarized macrophages (1 × 10^5^ cells in 200 μL medium containing 5% FBS) were placed in the lower chamber. After 12 hours of incubation, non-invading cells on the upper surface of the membrane were carefully removed and invading cells on the lower surface were fixed and stained with crystal violet.

### Statistical analysis

All statistical analyses were conducted using R software (version 4.2.3) and IBM SPSS Statistics (version 27.0.1). R packages included ConsensusClusterPlus, simplifyEnrichment (28), ggplot2, pheatmap, GSVA, ggpubr, survival, survminer, dplyr, Seurat, patchwork, and pROC. Pearson correlation, Student’s t-test, and one-way ANOVA were applied to assess group differences. Cox regression analysis was performed for survival analysis. *P* < 0.05 were considered statistically significant.

## Results

### M2 macrophages are spatially enriched and associated with poor prognosis in GBM

**Figure 1:**
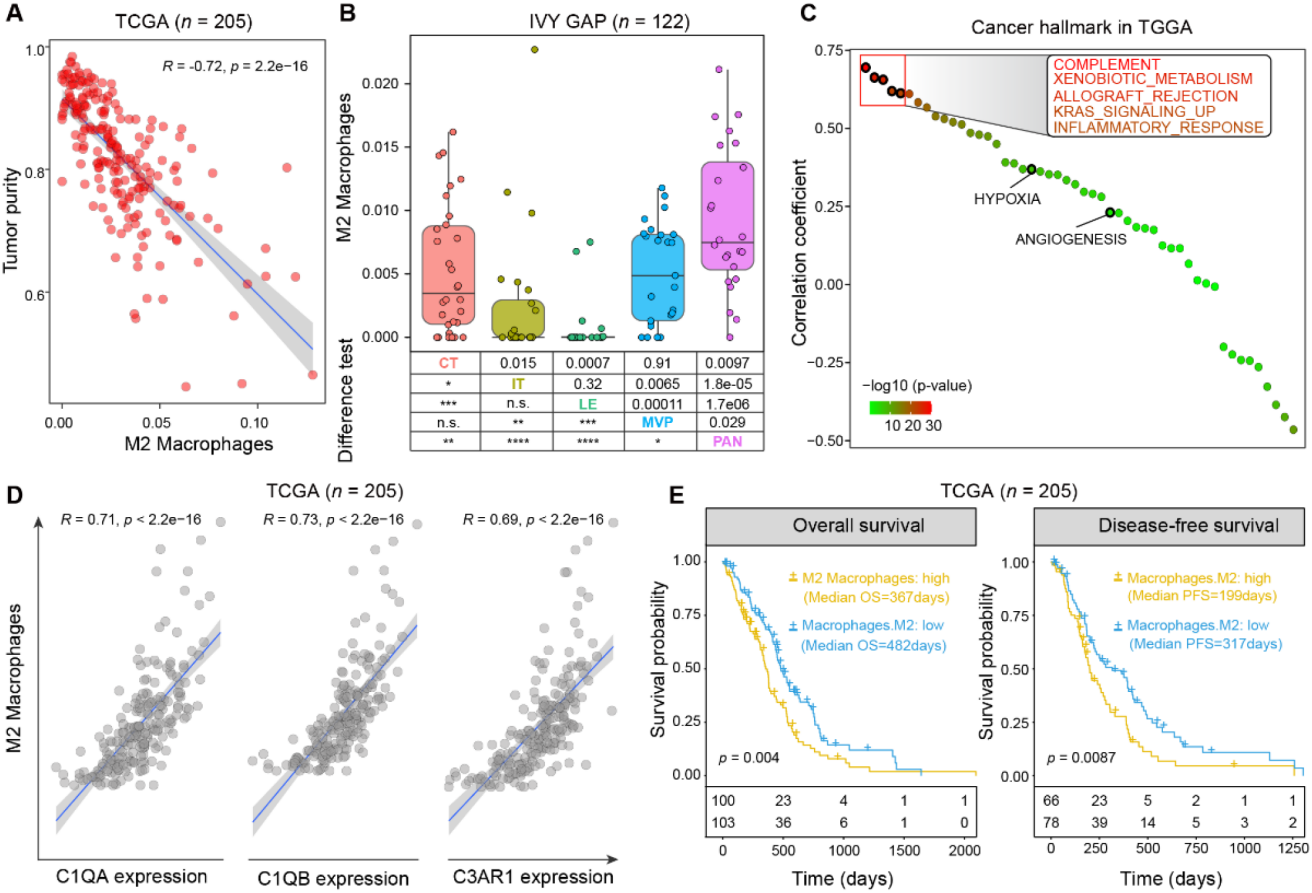
High M2 macrophage infiltration is associated with reduced tumor purity, spatial regions, complement, and poor prognosis in GBM. **(A)**, Scatter plot showing negative correlations between M2 macrophage scores (xCell) and tumor purity estimated by ESTIMATE in the TCGA cohorts. **(B)**, M2 macrophage scores in different GBM histological regions, including Cellular Tumor (CT), Infiltrating Tumor (IT), Leading Edge (LE), MicroVascular Proliferation (MVP), and Pseu- dopalisading cells Around Necrosis (PAN). **(C)**, Correlation analysis showing that M2 macrophage scores (xCell) are positively associated with the complement hallmark pathway estimated by ssGSEA in the TCGA cohort. **(D)**, Correlation analysis showing that M2 macrophage scores (xCell) are positively associated with the expression of key complement hallmark pathway genes, including C1QA, C1QB, and C3AR1. **(E)**, Kaplan–Meier survival curves comparing overall survival (left) and progression-free survival (right) between high and low M2 macrophage score groups in TCGA cohorts. Survival differences assessed by log-rank test (p values indicated).

To clarify the clinical and biological significance of M2 macrophage in glioblastoma (GBM), we quantified their infiltration using xCell-based deconvolution in two independent transcriptomic cohorts: TCGA (*n* = 205) and CGGA (*n* = 74). In both cohorts, higher M2 macrophage scores were strongly correlated with reduced tumor purity (*R* = -0.72, *p* < 0.0001; CGGA: *R* = -0.73, *p* < 0.0001; **Figure 1A**; **Supplementary Figure S1A**), indicating preferential accumulation of these cells within the non-neoplastic tumor microenvironment (TME) at the expense of tumor cell proportion.

To explore their spatial context, we analyzed anatomically annotated transcriptional profiles from the Ivy Glioblastoma Atlas Project (Ivy GAP, *n* = 270). M2 macrophage scores were markedly elevated in microvascular proliferation (MVP) and pseudopalisading cells around necrosis (PAN), two histological niches strongly linked to angiogenesis, hypoxia and aggressive tumor behavior (**Figure 1B**). Consistent with these findings, these regions were also exhibited in anti-inflammatory and pro-angiogenic pathways (**Figure 1C**; **Supplementary Figure S1B**), suggesting that M2 macrophages may be recruited to these specialized niches to promote vascular remodeling and immune evasion.

Functionally, gene set enrichment analysis (GSEA) further revealed that M2 macrophage infiltration positively correlated with pathways involved in complement activation (e.g., *C1QA*, *C1QB*, *C3AR1*) and immune modulation, including *inflammatory response*, *interferon gamma response*, and *TNFα signaling via NF-κB* (**Figure 1D**; **Supplementary Figures S1C–E**). These results indicate that beyond supporting angiogenesis, M2 macrophages actively shape an immunosuppressive microenvironment that suppresses anti-tumor immunity and facilitating tumor immune evasion.

Survival analyses further underscored the clinical relevance of these findings. Across both the TCGA and CGGA cohorts, patients with high M2 macrophage infiltration exhibited significantly shorter overall survival (OS) and progression-free survival (PFS) compared with those with low infiltration (TCGA: OS, *P* = 0.004; PFS, *P* = 0.0087; CGGA: OS, *P* = 0.033; PFS, *P* = 0.039). Specifically, in the TCGA cohort, the median OS was 367 days in the M2-high group versus 482 days in the M2-low group, with corresponding median PFS of 199 days and 317 days, respectively. Consistent results were observed in the CGGA cohort, where patients with high M2 infiltration had a median OS of 379 days compared with 591 days in the low-infiltration group, and median PFS of 259 days versus 550 days (**Figure 1E**; **Supplementary Figure S1F****).**Collectively, these findings demonstrate that M2 macrophages are spatially enriched in aggressive tumor niches, where they drive an immunosuppressive and pro-tumoral microenvironment, ultimately contributing to poor clinical outcomes in patients with GBM.

### M2 macrophage–related gene signatures define three prognostically relevant immune subtypes in GBM

**Figure 2:**
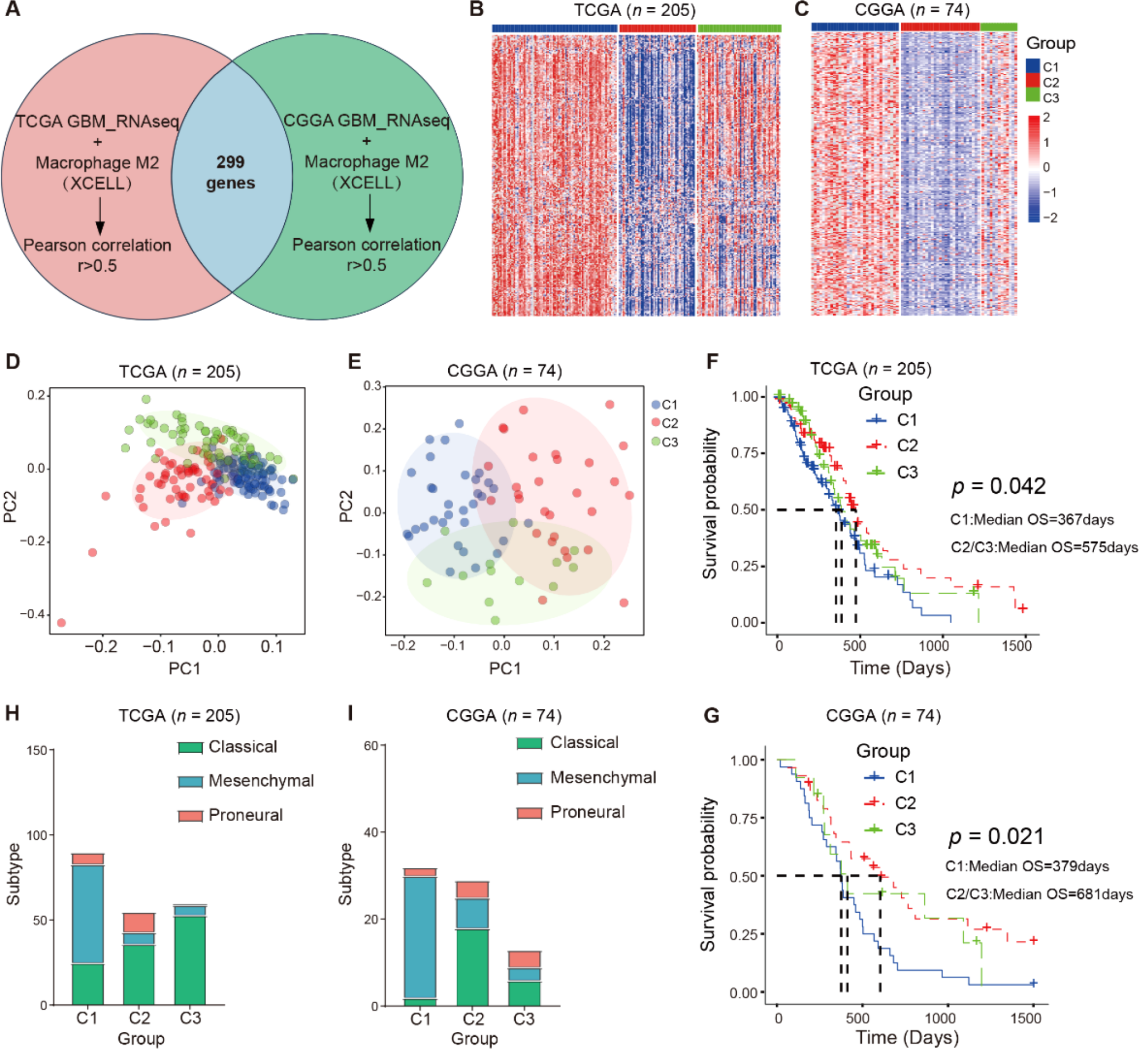
Identification and characterization of immune subtypes based on M2 macrophage–associated genes in GBM. **(A)**, Venn diagram showing 299 genes that were highly positively correlated with Macrophages_M2 scores (*R* > 0.5 & *p* < 0.05) in both TCGA and CGGA cohorts. **(B)**, Unsupervised consensus clustering based on the 299 M2-related genes identified three immune subtypes (C1, C2, and C3) in the TCGA cohort. **(C)**, A random forest classifier trained on TCGA data was applied to classify CGGA samples into the same three immune subtypes. **(D, E)**, Principal component analysis (PCA) of gene expression profiles showing clear separation among the three immune subtypes in both TCGA and CGGA cohorts. **(F, G)**, Kaplan–Meier survival curves comparing overall survival (OS) among the immune subtypes in TCGA and CGGA cohorts. Patients in the C1 group exhibited the worst prognosis. **(H–I)**, Distribution of molecular subtypes (Classical, Mesenchymal, Proneural) across the immune subtypes, with the mesenchymal subtype significantly enriched in the C1 group.

To characterize the immune heterogeneity of GBM, we first performed Pearson correlation analysis to identify genes strongly correlated with the M2 macrophages infiltration (*R* > 0.5 and *p* < 0.05) in both TCGA and CGGA cohorts. This analysis yielded 299 overlapping M2 macrophage-related genes (**Figure 2A**). Using these genes, unsupervised consensus clustering of the TCGA cohort stratified patients into three robust immune subtypes (C1, C2, and C3) (**Figure 2B**). A random forest classifier trained on the TCGA cohort was subsequently applied to the CGGA cohort, confirming the reproducibility of the three-subtype classification (TCGA: C1, *n* = 90; C2, *n* = 55; C3, *n* = 60; CGGA: C1, *n* = 32; C2, *n* = 29; C3, *n* = 13) (**Figure 2C**).

Principal component analysis (PCA) demonstrated clear separation among the three immune subtypes in both datasets (**Figures 2D, E**), supporting the robustness of the clustering. Survival analysis revealed that patients in the C1 subtype exhibited significantly short overall survival compared with those in the C2 and C3 subtypes (TCGA: *p* = 0.042; CGGA: *p* = 0.021) (**Figures 2F, G**), Specifically, in the TCGA cohort, patients classified as the C1 subtype had a median overall survival of 367 days, markedly shorter than the 575 days observed in the combined C2/C3 group. Consistent results were obtained in the CGGA cohort, where the median overall survival was 379 days for C1 patients compared with 681 days for those in the C2/C3 group, indicating that the C1 immune subtype is associated with the poorest prognosis.

Further molecular profiling revealed that mesenchymal transcriptional subtype was enriched within the C1 immune subtype (**Figures 2H, I**), suggesting that the high M2 macrophage activity is linked to mesenchymal transition and aggressive tumor biology.

Collectively, these findings demonstrate that M2 macrophage-related gene signature define three biologically and clinically distinct immune subtypes of GBM, providing a framework for understanding immune heterogeneity and informing the development of subtype-specific therapeutic strategies.

### C1 subtype exhibits an immune-infiltrated but immunosuppressive microenvironment

**Figure 3:**
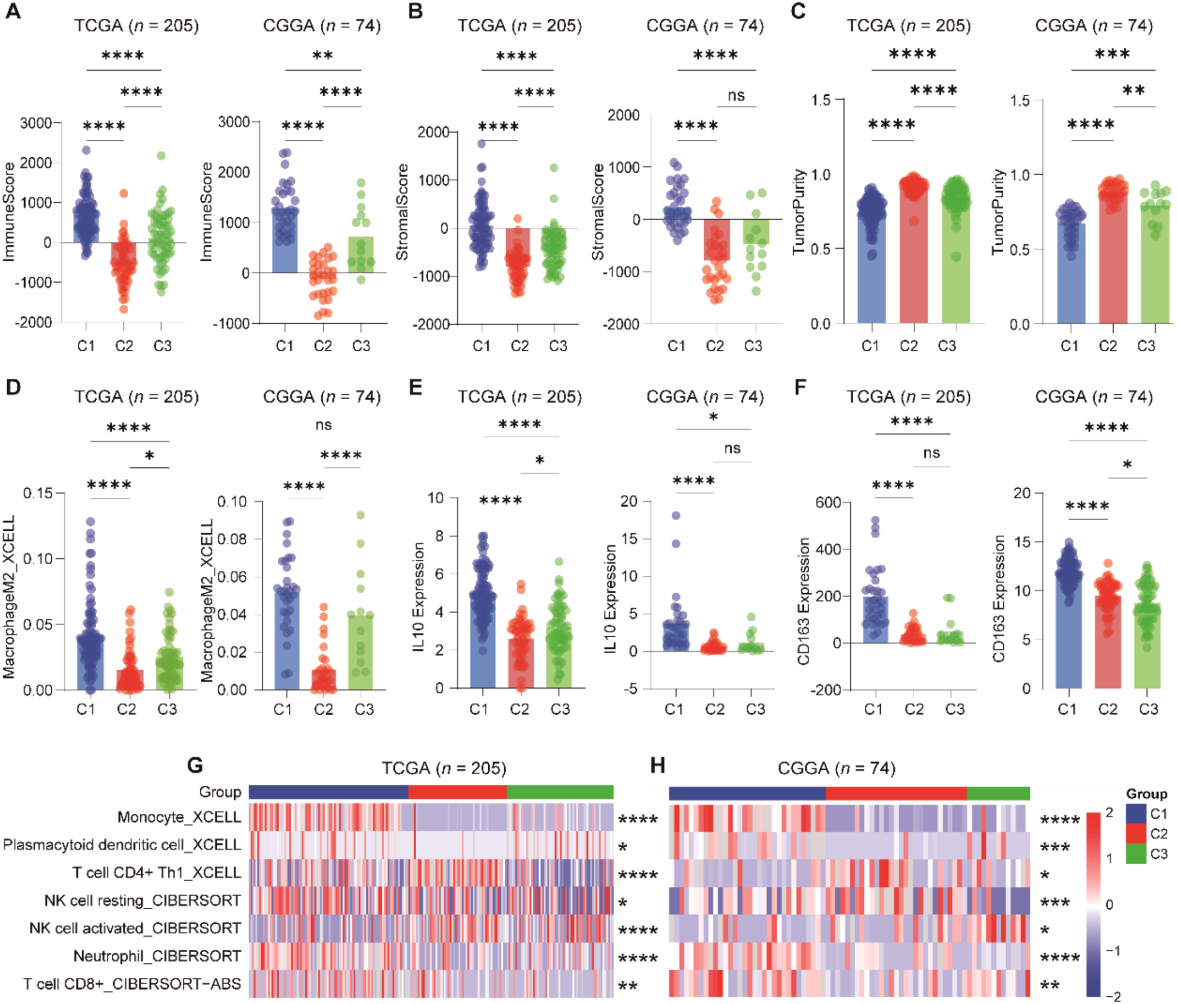
Immunological characterization of the C1–C3 subtypes in GBM. **(A–C)**, ESTIMATE analysis showing ImmuneScore, StromalScore, and tumor purity across the three immune subtypes in TCGA and CGGA cohorts. The C1 subtype exhibited the highest immune and stromal scores and the lowest tumor purity, whereas C2 displayed the opposite trend. **(D–F)**, Comparison of Macrophages_M2 scores and expression levels of canonical M2 markers (CD163 and IL10) among the three subtypes, showing highest M2 infiltration and marker expression in the C1 subtype. **(G, H)**, Infiltration of selected immune cell types across subtypes, estimated by xCell and CIBERSORT. The C1 subtype was enriched in immunosuppressive cells (monocytes, neutrophils, resting NK cells, exhausted CD8+ T cells) and depleted in antitumor effector cells (Th1 CD4+ T cells, activated NK cells). *p<0.05, **p<0.01; ***p<0.001; ****p<0.0001.

To better characterize the tumor microenvironment (TME) across M2 macrophage-base subtype, we applied the ESTIMATE algorithm to calculate immune score, stromal score, and tumor purity in both the TCGA and CGGA cohorts. In the TCGA cohort, the C1 subtype–which showed the poorest prognosis–exhibited the highest immune and stromal scores and markedly reduced tumor purity. Conversely, the C2 subtype, linked to the most favorable outcomes, displayed the lowest immune and stromal scores and the highest tumor purity. These patterns were consistently reproduced in the CGGA cohort (**Figures 3A–C**), confirming the robustness of subtype-specific microenvironmental features.

Importantly, the elevated immune and stromal scores in C1 did not indicate effective antitumor immunity. Instead, this subtype reflected a profoundly immunosuppressive TME dominated by M2-like tumor-associated macrophages (TAMs). Quantitative analysis revealed that the C1 subtype harbored the highest levels of M2 macrophage infiltration and elevated expression of canonical M2 markers, including *CD163* and *IL10* (**Figures 3D–F**).

Immune cell composition profiling further highlighted the tumor-supportive and dysfunctional nature of the C1 immune landscape. This subtype was enriched with monocytes, neutrophiles, resting NK cells, and a population of CD8+ T cells with an exhausted phenotype (**Figures 3G, H**). In contrast, effector cell populations with recognized antitumor activity–such as Th1-polarized CD4+ T cells and activated NK cells–were depleted in C1 and relatively enriched in the C2 subtype.

Collectively, these findings indicate that the C1 subtype represents an immune-infiltrated but immunosuppressive TME, where high immune cell content is driven by dysfunctional or tumor-supportive subsets rather than effective antitumor effectors. This immunological architecture, likely orchestrated by M2-polarized macrophages, may underlie the aggressive clinical behavior of C1 tumors and highlight the need for therapeutic strategies targeting immunosuppressive myeloid populations.

### Enrichment analysis reveals immune response-associated but potentially immunosuppressive signatures in the C1 subtype

**Figure 4:**
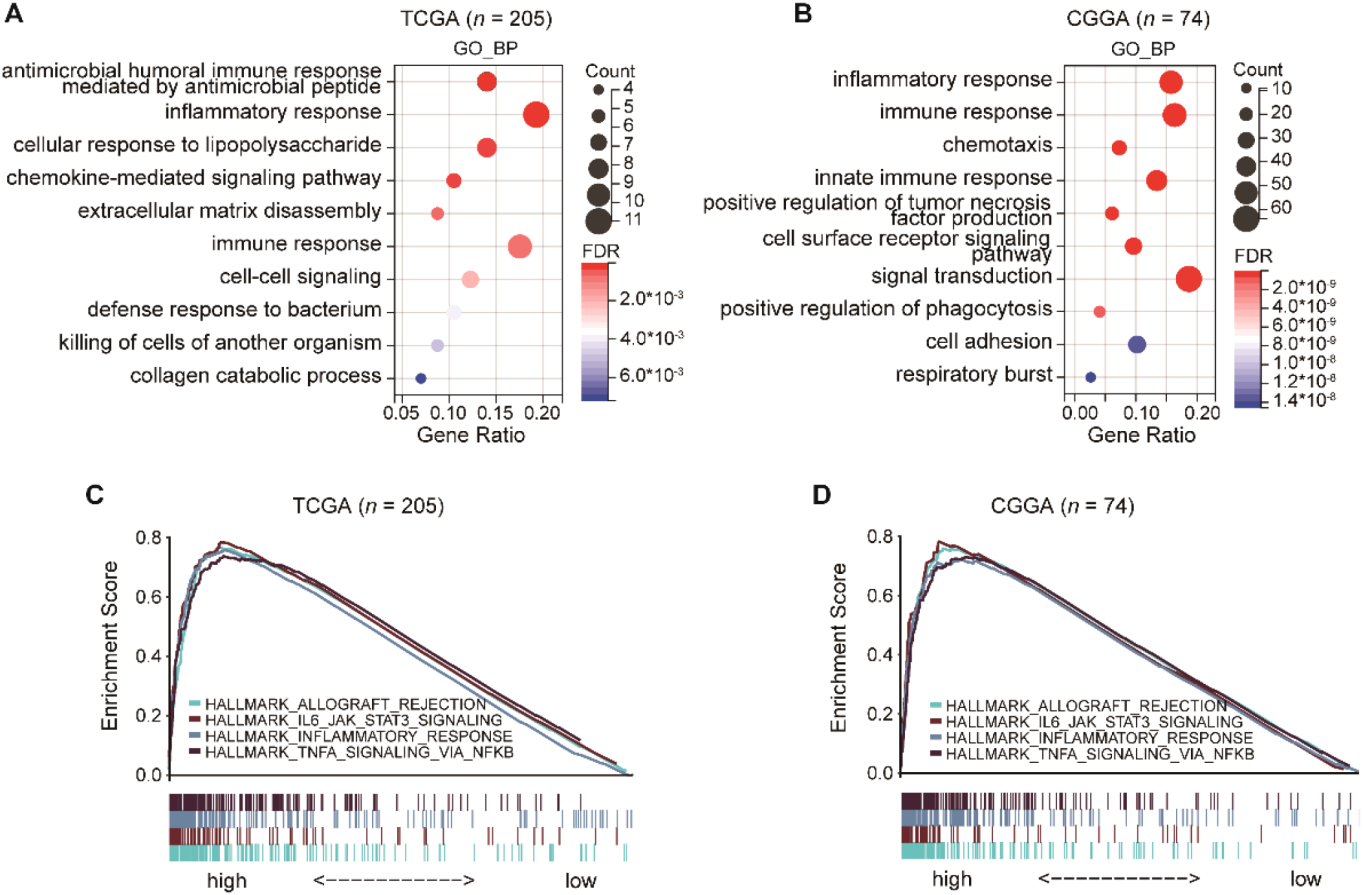
Functional enrichment analysis of genes upregulated in the C1 subtype. **(A, B)**, DAVID functional enrichment analysis of C1-upregulated genes (log_2_FC > 1, *p* < 0.05), highlighting immune-related biological processes. **(C, D)**, Gene set enrichment analysis (GSEA) based on CGGA and TCGA datasets showing significant enrichment of hallmark immune-related pathways in the C1 subtype, including Allograft Rejection, IL6_JAK_STAT3 Signaling, Inflammatory Response, and TNFA Signaling via NF-κB.

To explore the molecular underpinnings of the distinct tumor microenvironment observed in the C1 subtype, we performed functional enrichment analysis on genes significantly upregulated in C1 compared to C2 and C3 (log_2_FC > 1, *p* < 0.05). This analysis revealed significant enrichment of immune-related biological processes, such as *inflammatory response* and *immune response* (**Figures 4A, B**), indicating that the C1 subtype is characterized by a strong immune response–associated signature.

Consistently, Gene Set Enrichment Analysis (GSEA) in both the TCGA and CCGA cohorts demonstrated that C1-upregulated genes were significantly enriched in hallmark pathways, including *allograft rejection*, *IL6-JAK-STAT3 signaling*, *inflammatory response*, and *TNFα signaling via NF-κB* (**Figures 4C, D**). These pathways are well known to drive chronic inflammation, cytokine signaling, and immune cell recruitment–processes that often foster immune dysregulation rather that effective antitumor immunity.

Taken together, these findings suggest that although the C1 subtype exhibits a transcriptomic signature heightened immune responses, this activity primarily reflects an inflammation-driven, immunosuppressive microenvironment. This is consistent with the high abundance of M2 macrophages and dysfunctional effector immune subsets identified in our cellular infiltration analyses, underscoring the immune-evasive nature of the C1 subtype.

### XGBoost-LASSO–derived macrophage signature identified a high-risk, immunosuppressive C1 subtype in glioma

**Figure 5:**
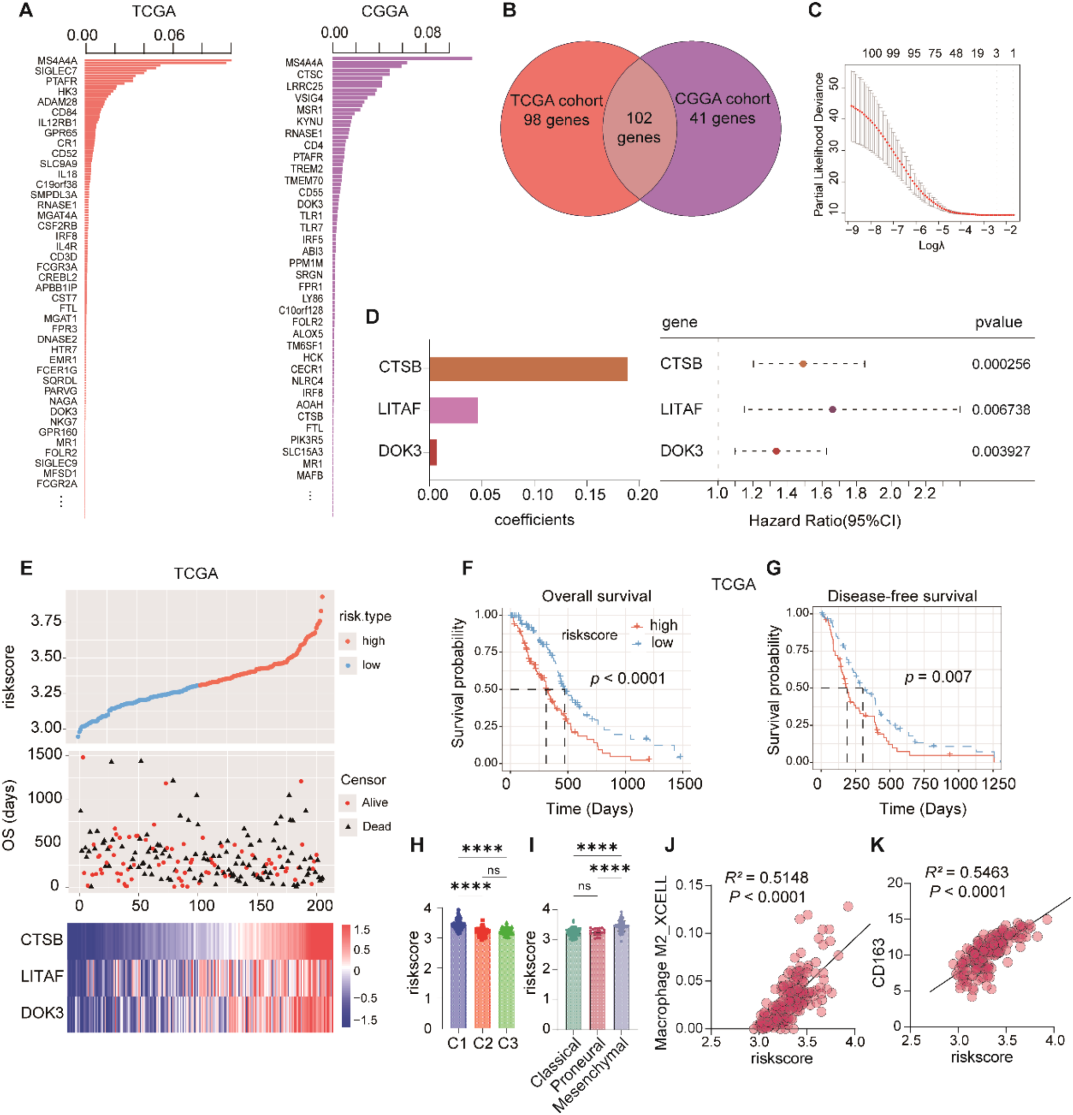
Construction of a macrophage-related risk score model to identify the C1 immunosuppressive subtype. **(A, B)**, Feature importance analysis by XGBoost in the TCGA and CGGA cohorts, identifying 200 and 143 M2-related genes with non-zero gain values, respectively. **(C)**, Venn diagram showing 102 overlapping genes with non-zero gain across both datasets. **(D)**, LASSO regression analysis of the 102 genes identified three key genes (*CTSB*, *LITAF*, and *DOK3*) used to construct the risk score model. **(E)**, Using the three candidate genes to group the risk scores, it was observed that the high-risk group (orange) had a poorer prognosis compared to the low-risk group (cyan). **(F, G)**, Kaplan-Meier survival analysis demonstrated that higher risk scores were associated with shorter OS and PFS. **(H)**, Comparison of risk scores among the three immune subtypes (C1-C3). **(I)**, Comparison of risk scores across GBM molecular subtypes, with the highest scores observed in the mesenchymal subtype. **(J, K)**, Correlation of the risk score with M2 macrophage infiltration **(J)** and *CD163* expression **(K)**. ****p<0.0001.

To pinpoint genes most closely associated with the C1 subtype and macrophage-driven immunosuppression, we leveraged the 299 macrophage-related genes previously identified as strongly correlated with M2 macrophage infiltration. Using these candidate features, we constructed a XGBoost-based classifier in both the TCGA and CGGA cohorts. The models identified 200 genes with non-zero gain values in TCGA and 143 genes in CGGA (**Figure 5A**). In the XGBoost framework, gain value represents the average performance improvement contributed by a feature when splitting decision nodes; thus, genes with non-zero gain values were considered important contributors to identifying the C1 subtype classification. Notably, 102 overlapping genes were consistently identified across both datasets (**Figure 5B**), underscoring their stable predictive utility in independent cohorts.

To further refine these candidates, we performed LASSO regression analysis to the 102 overlapping genes (**Figure 5C**), which yielded a concise three-gene macrophage signature composed of *CTSB*, *LITAF*, and *DOK3*, with regression coefficients of 0.18895, 0.04699, and 0.00799, respectively (**Figure 5D**). Based on these coefficients, we calculated a composite macrophage-related risk model for each patient and stratified individuals within the C1 subtype in both cohorts into high- and low-risk groups according to the median score. Patients in the high-risk group exhibited significantly higher mortality than those in the low-risk group (**Figure 5E****).**

Kaplan-Meier survival analyses demonstrated that patients with high-risk scores exhibited significantly shorter overall survival (OS) and progression-free survival (PFS) (**Figures 5F, G**). Specifically, in the TCGA cohort, the median OS was 312 days in the risk score-high group, compared with 474 days in the risk score-low group, while the corresponding median PFS values were 190.2 days and 303.6 days, respectively. In addition, the risk score was significantly elevated in the C1 subtype compared with the C2 and C3 subtypes (**Figure 5H**) and was also increased in the mesenchymal transcriptional subtype (**Figure 5I**), indicating a strong association between the risk score and more aggressive tumor phenotypes.

Correlation analyses further revealed that the macrophage-based risk score was positively associated with M2 macrophage infiltration (*R* = 0.72, *p* < 0.0001) (**Figure 5J**) and strongly correlated with the canonical M2 marker *CD163* (*R* = 0.75, *p* < 0.0001); (**Figure 5K**). These findings indicate that the risk score captures not only macrophage abundance but also the immunosuppressive activity of the tumor microenvironment.

Importantly, these results were independently validated in the CGGA cohort (**Supplementary Figures S2A–G**). In this validation dataset, patients in the risk score-high group exhibited a median OS of approximately 289 days, which was markedly shorter than the approximately 610 days observed in the risk score-low group; the corresponding median PFS values were approximately 259 days and 550 days, respectively. The consistent survival differences observed across independent cohorts further demonstrate the robustness and reproducibility of this macrophage-associated risk model.

### High risk score reflects an immune-infiltrated but immunosuppressive tumor microenvironment

**Figure 6:**
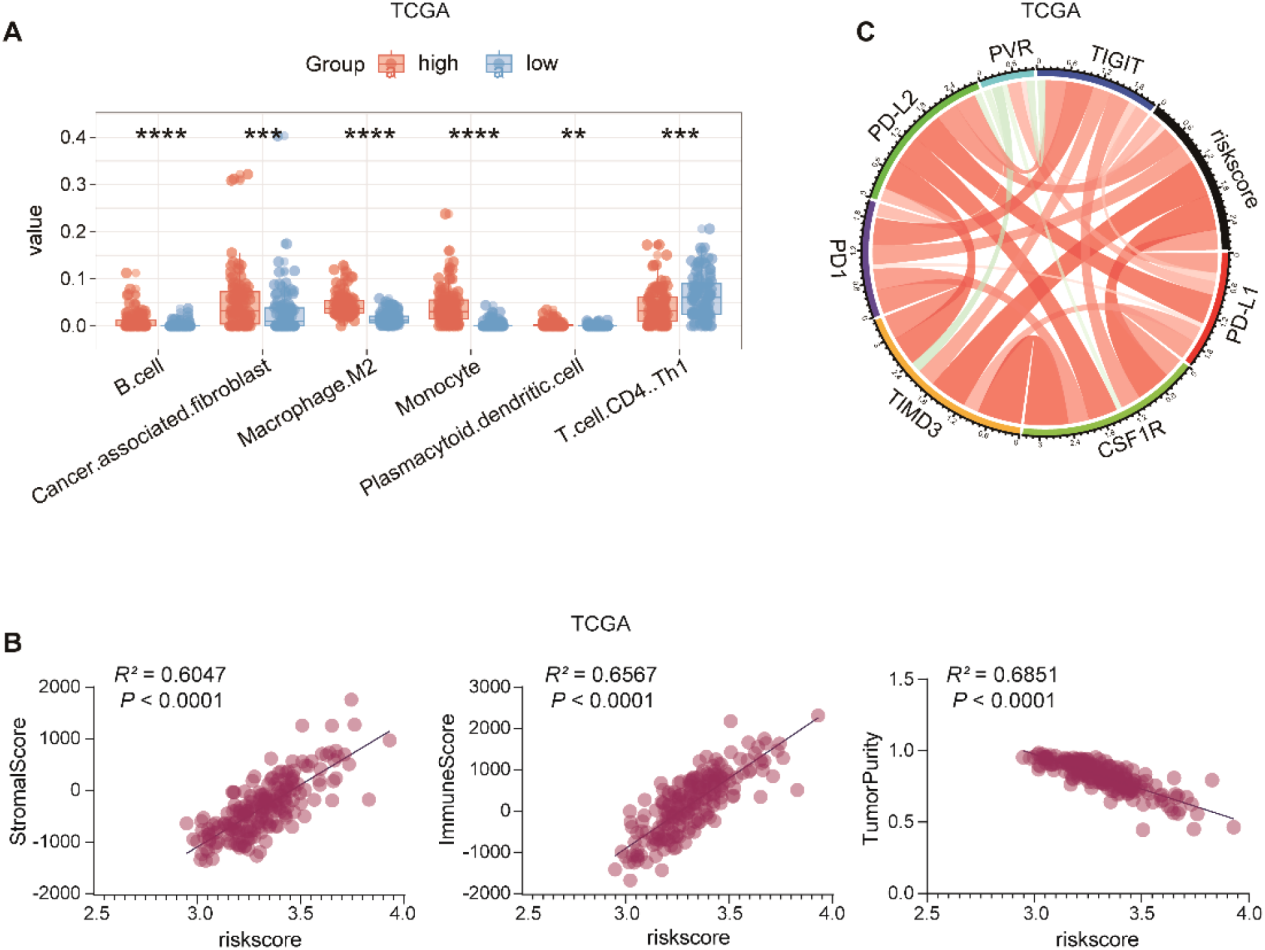
The association between the risk score and immune microenvironment characteristics in the TCGA cohort. **(A)**, Immune cell infiltration levels in high- and low-risk groups were assessed using the xCell algorithm. The high-risk group showed significantly elevated levels of several immunosuppressive cell types, including M2 macrophages, **(B)** cells, monocytes, cancer-associated fibroblasts (CAFs), and plasmacytoid dendritic cells (pDCs), while CD4^+^ Th1 cells were more abundant in the low-risk group. **(B)**, Correlation between risk score and ESTIMATE-derived stromal score, immune score, and tumor purity. **(C)**, Expression levels of immune checkpoint molecules (*TIMD3*, *CSF1R*, *PD-1*) are positively correlated with the risk score. **p<0.01; ***p<0.001; ****p<0.0001.

To elucidate the biological significance of the macrophage-based risk score within the TME, we analyzed immune cell composition using the xCell algorithm in both the TCGA and CGGA cohorts. The high-risk group exhibited significantly elevated infiltration of B cells, cancer-associated fibroblasts, M2 macrophages, monocytes, and plasmacytoid dendritic cells (**Figure 6A**; **Supplementary Figure S3A**)–cell populations widely implicated in immune suppression and tumor progression (29, 30). In contrast, the low-risk group exhibited higher levels of CD4^+^ Th1 T cells, a subset known for potent antitumor activity (31), suggesting that these cells may contribute to more active immune response in this subgroup.

We next assessed the overall immune and stromal contexture using the ESTIMATE algorithm. Both stromal and immune score were positively correlated with the risk score, whereas tumor purity showed a negative correlation (**Figure 6B**; **Supplementary Figure S3B**). These findings indicate that tumors with higher risk scores harbor greater infiltration of non-tumor components, consistent with a highly infiltrated yet immunologically suppressive TME.

To further explore potential mechanisms of immune suppression, we examined the relationship between the risk score and the expression of classical immune checkpoint molecules. Expression levels of *TIMD3*, *CSF1R*, and *PD-1* were significantly elevated in the high-risk group (**Figure 6C**; **Supplementary Figure S3C**), suggesting enhanced activation of immunosuppressive signaling pathways in these patients. This pattern aligns with the enrichment of M2 macrophages and other suppressive immune subsets, reinforcing the link between the high-risk signature and a dysfunctional, immunosuppressive microenvironment.

### DOK3 as a key regulator of M2 macrophage polarization

**Figure 7:**
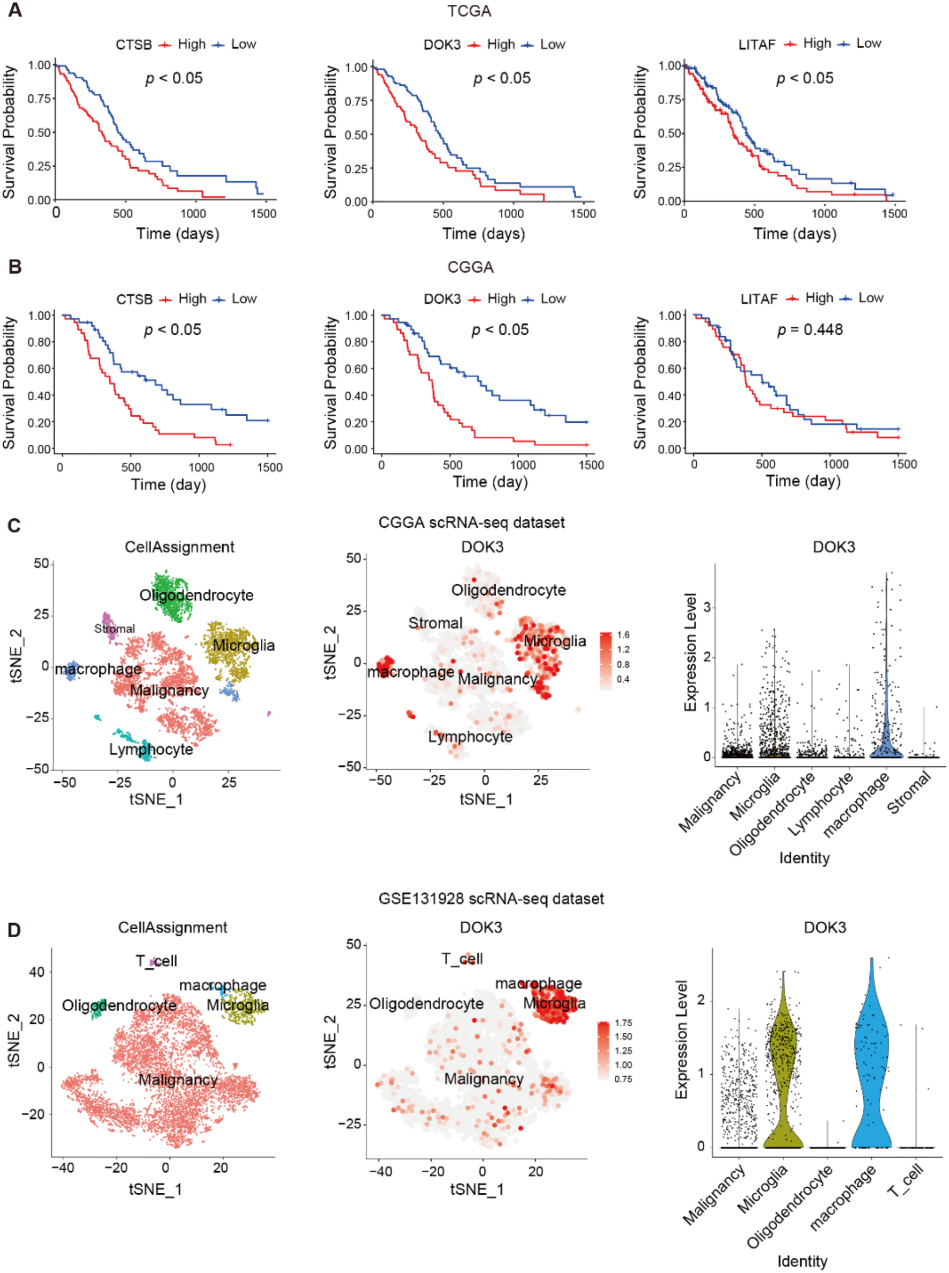
Expression patterns and prognostic relevance of CTSB, LITAF, and DOK3, with emphasis on DOK3 in myeloid populations. **(A, B)**, Kaplan-Meier survival analysis of *CTSB*, *LITAF*, and *DOK3* in TCGA **(A)** and CGGA **(B)** cohorts. High expression of *CTSB* and *DOK3* was associated with poor prognosis in both datasets, while LITAF showed prognostic value only in TCGA. **(C, D)**, Single-cell RNA-seq analysis of glioma samples from the CGGA scRNA-seq dataset **(C)** and GSE131928 scRNA-seq dataset **(D)**. UMAP plots depict cell clustering and gene expression of canonical myeloid markers (*CD68*, *TMEM119*, *CD163*) and DOK3, which is predominantly enriched in macrophages and microglia.

We next examined the three key genes–*CTSB*, *LITAF*, and *DOK3*–that constitute the macrophage-based risk model. In the TCGA dataset, high expression of all three genes was associated with worse prognosis (**Figure 7A**). In the CGGA dataset, elevated expression of *CTSB* and *DOK3* similarly correlated with poor survival, whereas *LITAF* expression showed no significant prognostic association (**Figure 7B**).

Given the significant positive correlation between the risk score, M2 macrophage infiltration, and *CD163* expression (**Figures 5J, K**), we further investigated the cellular localization of these genes using single-cell RNA sequencing (scRNA-seq) data from the GSE131928 and CGGA datasets. Clustering of tumor and microenvironmental cell populations–annotated canonical markers including *CD68* (macrophages), *TMEM119* (microglia), and *CD163* (M2 macrophages)–revealed that *DOK3* expression was predominantly restricted in microglia and macrophages (**Figures 7C, D**). In contrast, *CTSB* and *LITAF* exhibited broad, non-specific expression across multiple cell types, including malignant cells, stromal cells, lymphocytes, and oligodendrocytes (**Supplementary Figures S4A–D**) suggesting that their functional relevance may not be limited to macrophage functions.

These findings highlight *DOK3* as the most cell-type-specific gene with the risk signature and suggest that it may act as a regulator of M2 macrophage polarization. This polarization likely facilitates the establishment of an immunosuppressive tumor microenvironment, promoting immune evasion and supporting the malignant progression of gliomas.

Building on these findings, we further evaluated the histological and immunological features associated with DOK3 expression at the level of the tumor microenvironment. As shown in **Supplementary Figures S5A, B**, across both the TCGA and CGGA cohorts, DOK3 expression was positively correlated with immune and stromal scores, while negatively correlated with tumor purity, indicating that tumors with high DOK3 expression are characterized by a pronounced enrichment of non-tumor components, including immune and stromal cells. This microenvironmental profile is highly consistent with the mesenchymal phenotype enriched in the C1 immune subtype. Accordingly, DOK3 expression was significantly higher in the mesenchymal subtype than in the classical and proneural subtypes (**Supplementary Figures S5C, D**). Concurrently, immune cell infiltration patterns showed a similar distribution, with macrophage infiltration scores markedly elevated in the mesenchymal subtype (**Supplementary Figures S5E, F**). Together, these results further support a close association between DOK3 expression, immune cell (particularly macrophage) enrichment, and mesenchymal tumor characteristics.

### DOK3 knockdown inhibits M2 macrophage polarization and suppressed glioma cell migration

**Figure 8:**
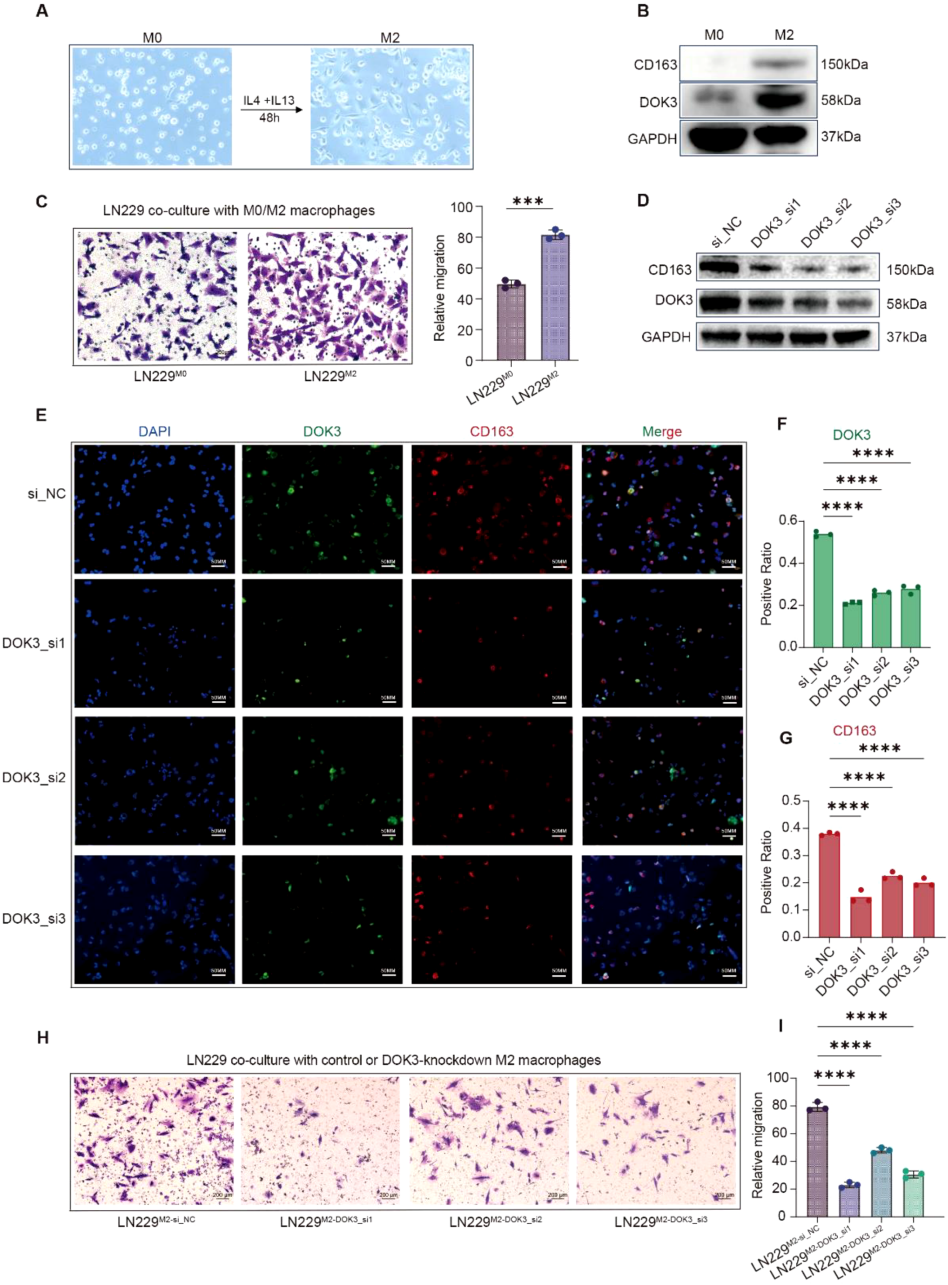
Validation of the functional involvement of DOK3 in macrophage polarization and glioma cell migration. **(A)**, Morphological changes of THP-1–derived macrophages during M0-to-M2 polarization following PMA treatment and IL-4/IL-13 stimulation. **(B)**, Western blot analysis showing increased expression of CD163 and DOK3 during M2 macrophage polarization. **(C)**, Transwell invasion assay showing enhanced invasiveness of LN229 glioma cells following co-culture with M2 macrophages. **(D)**, Western blot confirming DOK3 knockdown in M2 macrophages and the associated reduction in CD163 expression. **(E–G)**, Immunofluorescence staining showing decreased expression of DOK3 and CD163 in DOK3-silenced M2 macrophages. **(H, I)**, Transwell invasion assays demonstrating reduced LN229 cell invasiveness following culture with conditioned media from DOK3-silenced M2 macrophages. ***p<0.001; ****p<0.0001.

To experimentally validate the role of DOK3 in macrophage polarization and tumor progression, we performed a series of cell-based functional assays using THP-1 cells, a well-established *in vitro* model for human monocyte–macrophage biology. THP-1 cells were first treated with phorbol 12-myristate 13-acetate (PMA) to induce differentiation into M0 macrophages, followed by stimulation with IL-4 and IL-13 for 48 hours to drive M2 polarization. This polarization process was initially confirmed by the characteristic morphological transition from round to spindle-shaped cells (**Figure 8A**). Western blot analysis further demonstrated that during the transition from M0 to M2 macrophages, the protein expression levels of the M2 marker CD163 and DOK3 were both markedly increased (**Figure 8B**), indicating a close association between DOK3 expression and the M2-polarized state. Subsequently, M0 or M2 macrophages were co-cultured with LN229 glioma cells. As shown in **Figure 8C**, co-culture with M2 macrophages resulted in a significant increase in LN229 cell invasiveness, demonstrating that M2-polarized macrophages promote an invasive phenotype in glioma. Gene silencing of *DOK3* in M2-polarized macrophages led to a marked reduction in *DOK3* expression and a significant decrease in the M2 marker *CD163*, as confirmed by Western blot analysis (**Figure 8D**). Immunofluorescence staining corroborated these findings, showing diminished expression of both *DOK3* and *CD163* in the knockdown group compared with controls (**Figures 8E–G**). These results indicate that *DOK3* is essential for the maintenance of the M2-polarized phenotype.

To assess the functional relevance of *DOK3*-mediated polarization in tumor biology, we co-cultured LN229 glioma cells with conditioned media derived from *DOK3*-silenced M2 macrophages. Compared to controls, glioma cells invasiveness was significantly attenuated in the knockdown group (**Figures 8H, I**), demonstrating that *DOK3*-mediated M2 polarization facilitates a tumor-promoting microenvironment that enhances glioma cell migration.

Collectively, these findings provide direct functional evidence that DOK3 promotes M2 macrophage polarization and contributes to glioma aggressiveness. Targeting *DOK3* in macrophages may therefore represent a promising therapeutic strategy to reprogram the tumor immune microenvironment and restain glioma progression.

## Discussion

Glioblastoma (GBM) remains one of the most lethal malignancies of the central nervous system, characterized by extreme heterogeneity, aggressive invasiveness, and resistance to standard therapies (32). Increasing evidence underscores the pivotal role of the TME in driving tumor progression and therapeutic resistance. Among the immune components, TAMs–particularly those polarized toward the M2 phenotype–are recognized as key facilitators of immune evasion, tumor growth, and treatment failure (33–35). In this study, we systematically dissected the immunological and molecular landscape of M2 macrophages in GBM and identified *DOK3* as a potential regulator of M2 polarization and glioma progression.

Using xCell-based immune deconvolution across TCGA and CGGA cohorts, we demonstrated that elevated M2 macrophage infiltration is associated with poor prognosis and reduced tumor purity, consistent with their recognized role in shaping an immunosuppressive microenvironment. Unsupervised clustering of 299 M2-related genes identified three immune subtypes (C1-C3), among which the C1 subtype exhibited the poorest prognosis and was enriched for the mesenchymal molecular subtype. This C1 subtype displayed a higher infiltrated but profoundly immunosuppressive microenvironment, characterized by enrichment of M2 macrophages, monocytes, and resting NK cells, along with depletion of cytotoxic immune populations such as Th1-polarized CD4^+^ T cells and activated NK cell. Although CD8^+^ T cells were numerically abundant in C1 tumors, their functional exhaustion likely rendered them ineffective, reflecting an “immune-high but functionally suppressed” state that has been increasingly recognized as a hallmark of immune dysfunction in GBM (31, 36–38). This dysfunctional immune architecture may drive immune escape, malignant progression, and resistance to therapy.

Through integrative machine learning combining XGBoost and LASSO modeling, we developed a macrophage-related risk score that stratifies patients by prognosis and immune contexture. Among the three key genes identified (*CTSB*, *LITAF*, and *DOK3*), *DOK3* emerged as the most cell-type–specific marker, with strong enrichment in microglia and macrophages and a robust correlation with the M2 marker *CD163*. Single-cell transcriptomic analysis confirmed its selective expression in myeloid-derived populations, supporting a macrophage-specific role. Functional experiments provided direct evidence for this: *DOK3* knockdown in THP-1–derived macrophages inhibited M2 polarization, reduced *CD163* expression, and suppressed the pro-invasive phenotype of glioma cells exposed to conditioned medium from M2 macrophages. Collectively, these findings demonstrate that *DOK3* promotes M2 polarization and contribute to the establishment of an immunosuppressive, tumor-promoting microenvironment in GBM.

These findings carry important translational implications. *DOK3* may serve not only as a prognostic biomarker but also a potential therapeutic target to reprogram the TME in GBM. Therapeutic strategies aimed at inhibiting *DOK3* or modulating macrophage polarization could restore antitumor immune activity and potentially enhance the efficacy of existing immunotherapeutic approaches, including checkpoint inhibitors and myeloid-targeted therapies, in patients with GBM.

Liu et al. demonstrated that DOK3 expression is strongly correlated with macrophage infiltration and poor prognosis in glioma (39), Moreover, Xu et al. provided a comprehensive pan-cancer analysis elucidating the immunogenomic landscape of DOK3 and its potential as a druggable target (40). In addition, evidence from other tumor types suggests that DOK3 is involved in the immunoregulatory functions of tumor-associated macrophages (TAMs). For example, studies in oral squamous cell carcinoma (OSCC) have shown that *Porphyromonas gingivalis* infection markedly upregulates DOK3 expression in TAMs and may influence macrophage differentiation and tumor recurrence through TNF- and MAPK-related signaling pathways, implicating DOK3 in tumor-associated inflammation and macrophage functional regulation (41). However, it should be noted that these studies primarily relied on bulk-tissue–level correlative analyses, or were confined to specific etiological factors or tumor contexts describing changes in DOK3 expression. To date, the functional role of DOK3 in M2 macrophage polarization has not been systematically elucidated in glioblastoma, nor has DOK3 been examined within an integrated immune stratification framework. In contrast, our study places DOK3 within a GBM-specific, M2 macrophage-centered immune stratification system, revealing its close association with an immunosuppressive microenvironment and poor clinical outcome at the population level, while single-cell transcriptomic analyses further confirm its cell-type–specific expression in microglia and macrophages. More importantly, we provide direct causal evidence from *in vitro* functional experiments, demonstrating that DOK3 regulates M2 macrophage polarization and promotes glioma cell invasiveness. Collectively, these findings extend previously observed “DOK3–TAM correlations” in other tumor types into a functionally defined “DOK3–M2 polarization–tumor progression” regulatory axis in glioblastoma.

The C1–C3 immune subtyping proposed in this study represents an unsupervised analytical strategy designed to systematically characterize the heterogeneity of the M2 macrophage–associated immune microenvironment in GBM and to identify a representative C1 subtype with pronounced immunosuppressive features and unfavorable prognosis. Based on the key molecular characteristics defining the C1 subtype, we further derived a simplified and quantitative high/low-risk model to enable a more intuitive and clinically actionable prognostic stratification. Overall, immune subtyping is more suitable for in-depth mechanistic investigations and comprehensive dissection of the immune microenvironment, whereas risk score models composed of a limited number of key genes offer greater operability and scalability in practical applications. The identification of DOK3 follows this hierarchical research framework, with its core value lying in bridging the global immune features captured by immune subtypes and functionally meaningful key molecules. Therefore, from a potential clinical translation perspective, these two research strategies demonstrate strong complementarity.

Despite the strengths of this study, including integrative multi-omics analyses and functional validation, several limitations should be acknowledged. The absence of *in vivo* validation limits our ability to fully assess the therapeutic potential and mechanistic pathways of *DOK3* in the complex TME. Moreover, while our data support a critical role of *DOK3* in macrophage polarization, the downstream signaling pathways and regulatory networks remain to be elucidated. Finally, the profound heterogeneity of GBM underscores the need for larger, multi-center studies and the integration of spatial single-cell multi-omics to better characterize immune interactions and uncover context-specific therapeutic vulnerabilities.

## Conclusion

**Figure 9:**
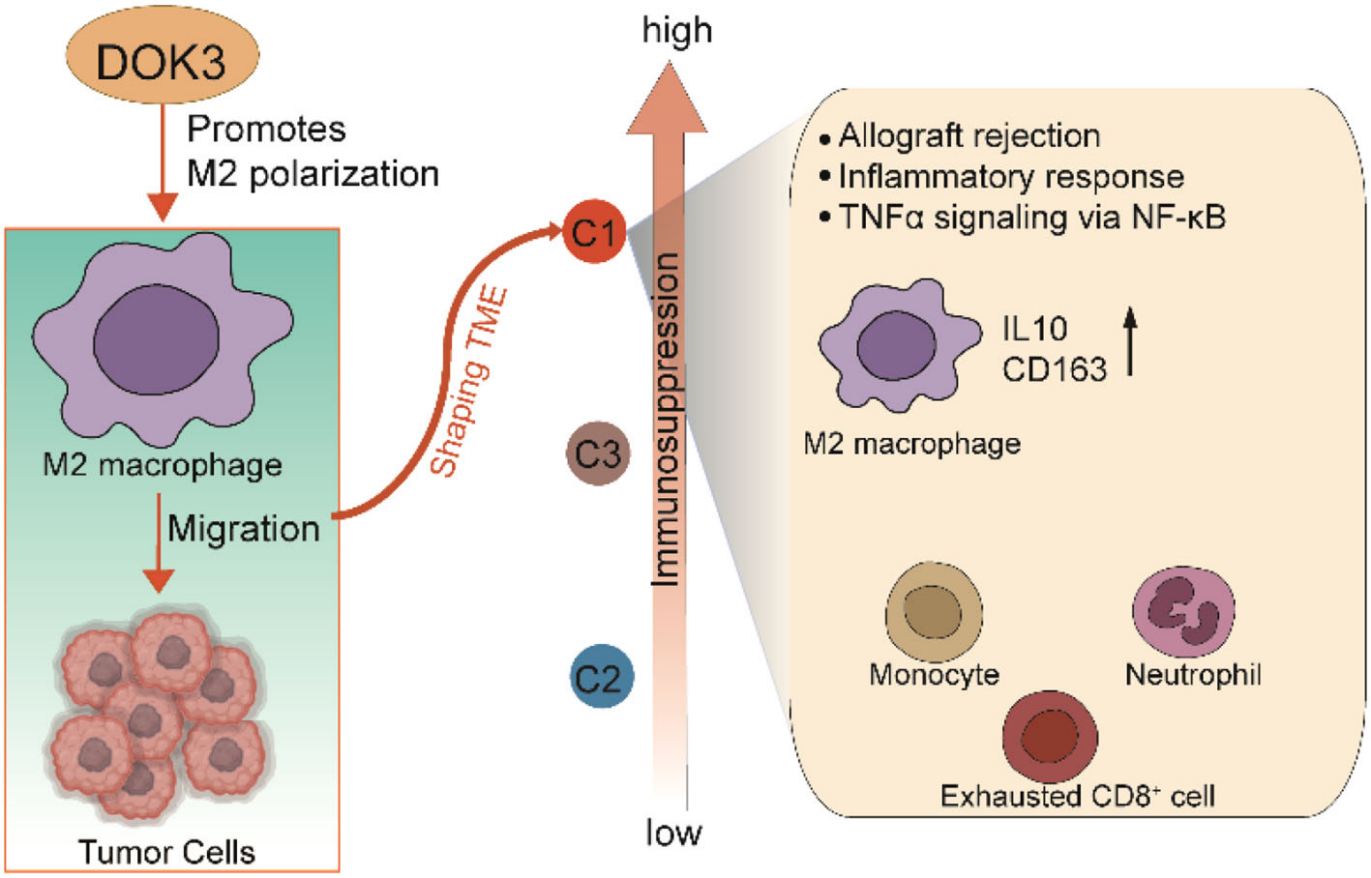
DOK3 can promote the polarization of macrophages toward the M2 pro-tumor phenotype, thereby participating in the construction of the immunosuppressive microenvironment of glioblastoma multiforme (GBM) and driving the malignant progression of gliomas.

This study comprehensively clarifies the characteristics of the immunosuppressive C1 subtype driven by M2 macrophages in glioblastoma multiforme (GBM), and confirms that DOK3 can promote the polarization of macrophages toward the M2 pro-tumor phenotype, thereby participating in the construction of the immunosuppressive microenvironment of GBM and driving the malignant progression of gliomas (**Figure 9**). Additionally, the risk model constructed based on macrophages provides a reliable basis for prognostic stratification and clinical therapeutic decision-making in GBM patients. These findings fully highlight the potential of DOK3 and macrophage reprogramming as therapeutic targets, which are expected to remodel the tumor immune microenvironment in GBM patients and further improve the clinical efficacy of immunotherapeutic strategies.

## Data Availability

The datasets presented in this study can be found in online repositories. The names of the repository/repositories and accession number(s) can be found below: The Cancer Genome Atlas (TCGA) (https://portal.gdc.cancer.gov/) and the Chinese Glioma Genome Atlas (CGGA) (http://www.cgga.org.cn/).

## References

[1] Koshy M Villano JL Dolecek TA Howard A Mahmood U Chmura SJ . Improved survival time trends for glioblastoma using the SEER 17 population-based registries. J Neuro-Oncol. (2012) 107:207–12. doi: 10.1007/s11060-011-0738-7, PMID: 21984115 PMC4077033

[2] Ostrom QT Price M Neff C Cioffi G Waite KA Kruchko C . CBTRUS statistical report: primary brain and other central nervous system tumors diagnosed in the United States in 2015-2019. Neuro-Oncology. (2022) 24:v1–v95. doi: 10.1093/neuonc/noac202, PMID: 36196752 PMC9533228

[3] Ostrom QT Shoaf ML Cioffi G Waite K Kruchko C Wen PY . National-level overall survival patterns for molecularly-defined diffuse glioma types in the United States. Neuro-Oncology. (2023) 25:799–807. doi: 10.1093/neuonc/noac198, PMID: 35994777 PMC10076944

[4] Gangoso E Southgate B Bradley L Rus S Galvez-Cancino F McGivern N . Glioblastomas acquire myeloid-affiliated transcriptional programs via epigenetic immunoediting to elicit immune evasion. Cell. (2021) 184:2454–2470.e26. doi: 10.1016/j.cell.2021.03.023, PMID: 33857425 PMC8099351

[5] Louis DN Perry A Wesseling P Brat DJ Cree IA Figarella-Branger D . The 2021 WHO classification of tumors of the central nervous system: a summary. Neuro-Oncology. (2021) 23:1231–51. doi: 10.1093/neuonc/noab106, PMID: 34185076 PMC8328013

[6] Mei Y Wang X Zhang J Liu D He J Huang C . Siglec-9 acts as an immune-checkpoint molecule on macrophages in glioblastoma, restricting T-cell priming and immunotherapy response. Nat Cancer. (2023) 4:1273–91. doi: 10.1038/s43018-023-00598-9, PMID: 37460871

[7] Zhao R Pan Z Qiu J Li B Qi Y Gao Z . Blocking ITGA5 potentiates the efficacy of anti-PD-1 therapy on glioblastoma by remodeling tumor-associated macrophages. Cancer Commun (Lon Eng). (2025) 45:677–701. doi: 10.1002/cac2.70016, PMID: 40084746 PMC12187582

[8] Wu L Wu W Zhang J Zhao Z Li L Zhu M . Natural coevolution of tumor and immunoenvironment in glioblastoma. Cancer Discov. (2022) 12:2820–37. doi: 10.1158/2159-8290.CD-22-0196, PMID: 36122307 PMC9716251

[9] Ge W Wu W . Influencing factors and significance of tumor-associated macrophage polarization in tumor microenvironment. Zhongguo Fei Ai Za Zhi = Chin J Lung Cancer. (2023) 26:228–37. doi: 10.3779/j.issn.1009-3419.2023.106.07, PMID: 37035885 PMC10106802

[10] Cao C Yin H Yang B Yue Q Wu G Gu M . Intra-operative definition of glioma infiltrative margins by visualizing immunosuppressive tumor-associated macrophages. Adv Sci (Weinheim Baden-Wurttemberg Germany). (2023) 10:e2304020. doi: 10.1002/advs.202304020, PMID: 37544917 PMC10558635

[11] Kureshi CT Dougan SK . Cytokines in cancer. Cancer Cell. (2025) 43:15–35. doi: 10.1016/j.ccell.2024.11.011, PMID: 39672170 PMC11841838

[12] Zheng C Wang J Zhou Y Duan Y Zheng R Xie Y . IFNα-induced BST2(+) tumor-associated macrophages facilitate immunosuppression and tumor growth in pancreatic cancer by ERK-CXCL7 signaling. Cell Rep. (2024) 43:114088. doi: 10.1016/j.celrep.2024.114088, PMID: 38602878

[13] Li J Wang K Yang C Zhu K Jiang C Wang M . Tumor-associated macrophage-derived exosomal LINC01232 induces the immune escape in glioma by decreasing surface MHC-I expression. Adv Sci (Weinheim Baden-Wurttemberg Germany). (2023) 10:e2207067. doi: 10.1002/advs.202207067, PMID: 37097629 PMC10265094

[14] Krneta T Gillgrass A Poznanski S Chew M Lee AJ Kolb M . M2-polarized and tumor-associated macrophages alter NK cell phenotype and function in a contact-dependent manner. J Leukocyte Biol. (2017) 101:285–95. doi: 10.1189/jlb.3A1215-552R, PMID: 27493241

[15] Ghiringhelli F Ménard C Terme M Flament C Taieb J Chaput N . CD4+CD25+ regulatory T cells inhibit natural killer cell functions in a transforming growth factor-beta-dependent manner. J Exp Med. (2005) 202:1075–85. doi: 10.1084/jem.20051511, PMID: 16230475 PMC2213209

[16] Groh V Wu J Yee C Spies T . Tumour-derived soluble MIC ligands impair expression of NKG2D and T-cell activation. Nature. (2002) 419:734–8. doi: 10.1038/nature01112, PMID: 12384702

[17] Batlle E Massagué J . Transforming growth factor-β Signaling in immunity and cancer. Immunity. (2019) 50:924–40. doi: 10.1016/j.immuni.2019.03.024, PMID: 30995507 PMC7507121

[18] Pyonteck SM Akkari L Schuhmacher AJ Bowman RL Sevenich L Quail DF . CSF-1R inhibition alters macrophage polarization and blocks glioma progression. Nat Med. (2013) 19:1264–72. doi: 10.1038/nm.3337, PMID: 24056773 PMC3840724

[19] Zhao Z Zhang KN Wang Q Li G Zeng F Zhang Y . Chinese glioma genome atlas (CGGA): A comprehensive resource with functional genomic data from chinese glioma patients. Genom Proteomics Bioinf. (2021) 19:1–12. doi: 10.1016/j.gpb.2020.10.005, PMID: 33662628 PMC8498921

[20] Zhao Z Meng F Wang W Wang Z Zhang C Jiang T . Comprehensive RNA-seq transcriptomic profiling in the Malignant progression of gliomas. Sci Data. (2017) 4:170024. doi: 10.1038/sdata.2017.24, PMID: 28291232 PMC5349247

[21] Puchalski RB Shah N Miller J Dalley R Nomura SR Yoon JG . An anatomic transcriptional atlas of human glioblastoma. Science. (2018) 360:660–3. doi: 10.1126/science.aaf2666, PMID: 29748285 PMC6414061

[22] Edgar R Domrachev M Lash AE . Gene Expression Omnibus: NCBI gene expression and hybridization array data repository. Nucleic Acids Res. (2002) 30:207–10. doi: 10.1093/nar/30.1.207, PMID: 11752295 PMC99122

[23] Li T Fu J Zeng Z Cohen D Li J Chen Q . TIMER2.0 for analysis of tumor-infiltrating immune cells. Nucleic Acids Res. (2020) 48:W509–w514. doi: 10.1093/nar/gkaa407, PMID: 32442275 PMC7319575

[24] Aran D Hu Z Butte AJ . xCell: digitally portraying the tissue cellular heterogeneity landscape. Genome Biol. (2017) 18:220. doi: 10.1186/s13059-017-1349-1, PMID: 29141660 PMC5688663

[25] Butler A Hoffman P Smibert P Papalexi E Satija R . Integrating single-cell transcriptomic data across different conditions, technologies, and species. Nat Biotechnol. (2018) 36:411–20. doi: 10.1038/nbt.4096, PMID: 29608179 PMC6700744

[26] Daigneault M Preston JA Marriott HM Whyte MK Dockrell DH . The identification of markers of macrophage differentiation in PMA-stimulated THP-1 cells and monocyte-derived macrophages. PloS One. (2010) 5:e8668. doi: 10.1371/journal.pone.0008668, PMID: 20084270 PMC2800192

[27] Martinez FO Gordon S Locati M Mantovani A . Transcriptional profiling of the human monocyte-to-macrophage differentiation and polarization: new molecules and patterns of gene expression. J Immunol. (2006) 177:7303–11. doi: 10.4049/jimmunol.177.10.7303, PMID: 17082649

[28] Gu Z Hubschmann D . simplifyEnrichment: A bioconductor package for clustering and visualizing functional enrichment results. Genomics Proteomics Bioinf. (2023) 21:190–202. doi: 10.1016/j.gpb.2022.04.008, PMID: 35680096 PMC10373083

[29] Shalapour S Font-Burgada J Di Caro G Zhong Z Sanchez-Lopez E Dhar D . Immunosuppressive plasma cells impede T-cell-dependent immunogenic chemotherapy. Nature. (2015) 521:94–8. doi: 10.1038/nature14395, PMID: 25924065 PMC4501632

[30] Kalluri R . The biology and function of fibroblasts in cancer. Nat Rev Cancer. (2016) 16:582–98. doi: 10.1038/nrc.2016.73, PMID: 27550820

[31] Kennedy R Celis E . Multiple roles for CD4+ T cells in anti-tumor immune responses. Immunol Rev. (2008) 222:129–44. doi: 10.1111/j.1600-065X.2008.00616.x, PMID: 18363998

[32] Lim M Xia Y Bettegowda C Weller M . Current state of immunotherapy for glioblastoma. Nat Rev Clin Oncol. (2018) 15:422–42. doi: 10.1038/s41571-018-0003-5, PMID: 29643471

[33] Quail DF Joyce JA . The microenvironmental landscape of brain tumors. Cancer Cell. (2017) 31:326–41. doi: 10.1016/j.ccell.2017.02.009, PMID: 28292436 PMC5424263

[34] Hambardzumyan D Gutmann DH Kettenmann H . The role of microglia and macrophages in glioma maintenance and progression. Nat Neurosci. (2016) 19:20–7. doi: 10.1038/nn.4185, PMID: 26713745 PMC4876023

[35] Chen Y Huo R Kang W Liu Y Zhao Z Fu W . Tumor-associated monocytes promote mesenchymal transformation through EGFR signaling in glioma. Cell Rep Med. (2023) 4:101177. doi: 10.1016/j.xcrm.2023.101177, PMID: 37652019 PMC10518634

[36] Vivier E Raulet DH Moretta A Caligiuri MA Zitvogel L Lanier LL . Innate or adaptive immunity? The example of natural killer cells. Science. (2011) 331:44–9. doi: 10.1126/science.1198687, PMID: 21212348 PMC3089969

[37] Wherry EJ . T cell exhaustion. Nat Immunol. (2011) 12:492–9. doi: 10.1038/ni.2035, PMID: 21739672

[38] Woroniecka K Chongsathidkiet P Rhodin K Kemeny H Dechant C Farber SH . T-cell exhaustion signatures vary with tumor type and are severe in glioblastoma. Clin Cancer Res. (2018) 24:4175–86. doi: 10.1158/1078-0432.CCR-17-1846, PMID: 29437767 PMC6081269

[39] Liu X Chen F Li W . Elevated expression of DOK3 indicates high suppressive immune cell infiltration and unfavorable prognosis of gliomas. Int Immunopharmacol. (2020) 83:106400. doi: 10.1016/j.intimp.2020.106400, PMID: 32193105

[40] Xu Z Cai L Jiang Q Xin Y Chen S Liu J . Pan-cancer analysis identified DOK3 as a novel biomarker for predicting prognosis and immunotherapy effectiveness. Dis Oncol. (2025) 16:2290. doi: 10.1007/s12672-025-04133-3, PMID: 41286270 PMC12748382

[41] Li CX Su Y Gong ZC Liu H . Porphyromonas gingivalis Activation of Tumor-Associated Macrophages via DOK3 Promotes Recurrence of Oral Squamous Cell Carcinoma. Med Sci Monitor. 21:e937126. doi: 10.12659/MSM.937126, PMID: 36210538 PMC9562789

