# Supplementary material for: M2 macrophage-based classification identifies DOK3 as a driver of pro-tumoral polarization and migration in glioblastoma
Source: Front Immunol. 2026 Jan 29;17:1725581. doi: 10.3389/fimmu.2026.1725581 (PMC12894355; doi:10.3389/fimmu.2026.1725581)
Supplement: Supplementary file 1 [file DataSheet1.pdf]

Supplementary Figure S1. High M2 macrophage infiltration is associated with reduced tumor purity, complement pathway activation, and poor prognosis in GBM in the CGGA cohort. **A,** Scatter plot showing a negative correlation between M2 macrophage scores (xCell) and tumor purity estimated by ESTIMATE in the TCGA cohort. **B,** Correlation analysis showing a positive association between M2 macrophage scores (xCell) and the Hallmark complement pathway estimated by ssGSEA. **C–D,** GSEA analysis showing enrichment of complement-related pathways in samples with high M2 macrophage infiltration. **E,** Correlation analysis showing a positive association between M2 macrophage scores (xCell) and the expression of key complement pathway genes, including *C1QA*, *C1QB*, and *C3AR1*. **F,** Kaplan–Meier survival curves comparing overall survival (left) and progression-free survival (right) between high and low M2 macrophage score groups in the CGGA cohort. Survival differences were assessed using the log-rank test (p values indicated).

Supplementary Figure S2. Construction of a macrophage-related risk score model to identify the C1 immunosuppressive subtype in CGGA cohort. A, Using the three candidate genes to group the risk scores, it was observed that the high-risk group (orange) had a poorer prognosis compared to the low-risk group (cyan). **B-C,** Kaplan-Meier survival analysis demonstrated that higher risk scores were associated with shorter OS and PFS. **D,** Comparison of risk scores among the three immune subtypes (C1-C3). **E,** Comparison of risk scores across GBM molecular subtypes, with the highest scores observed in the mesenchymal subtype. **F-G,** Correlation of the risk score with M2 macrophage infiltration (F) and CD163 expression (G).

Supplementary Figure S3. Validation of the immune microenvironment features in the CGGA cohort. A, Immune cell infiltration differences between high- and low-risk groups based on xCell analysis, consistent with the CGGA cohort. **B,** Correlation of the risk score with immune score, stromal score, and tumor purity as computed by ESTIMATE. **C,** Positive correlation between risk score and the expression of immune checkpoint genes *TIMD3*, *CSF1R*, and *PD-1*, confirming the immunosuppressive profile of high-risk tumors.

Supplementary Figure S4. CTSB and LITAF exhibit broad, non-specific expression across multiple cell types. A-D, Single-cell RNA-seq (scRNA-seq) analysis of glioma samples from the CGGA and GSE131928 datasets. UMAP plots show cell clustering and the expression of canonical myeloid markers (*CD68*, *TMEM119*, *CD163*) as well as *CTSB* (A, C) and *LITAF* (B, D), demonstrating that *CTSB* and *LITAF* are expressed across multiple cell types.

Supplementary Figure S5. Association of DOK3 expression with tumor microenvironment features and molecular subtypes. A–B, Correlation analyses showing the relationship between DOK3 expression and immune score, stromal score, and tumor purity in the TCGA (A) and CGGA (B) cohorts. DOK3 expression is positively correlated with immune and stromal scores and negatively correlated with tumor purity. **C–D,** Comparison of DOK3 expression levels across molecular subtypes, demonstrating significantly higher expression in the mesenchymal subtype

compared with the classical and proneural subtypes in the TCGA (C) and CGGA (D) cohorts. **E–F,** Distribution of macrophage infiltration scores across molecular subtypes in the TCGA (E) and CGGA (F) cohorts, showing significantly increased macrophage infiltration in the mesenchymal subtype.

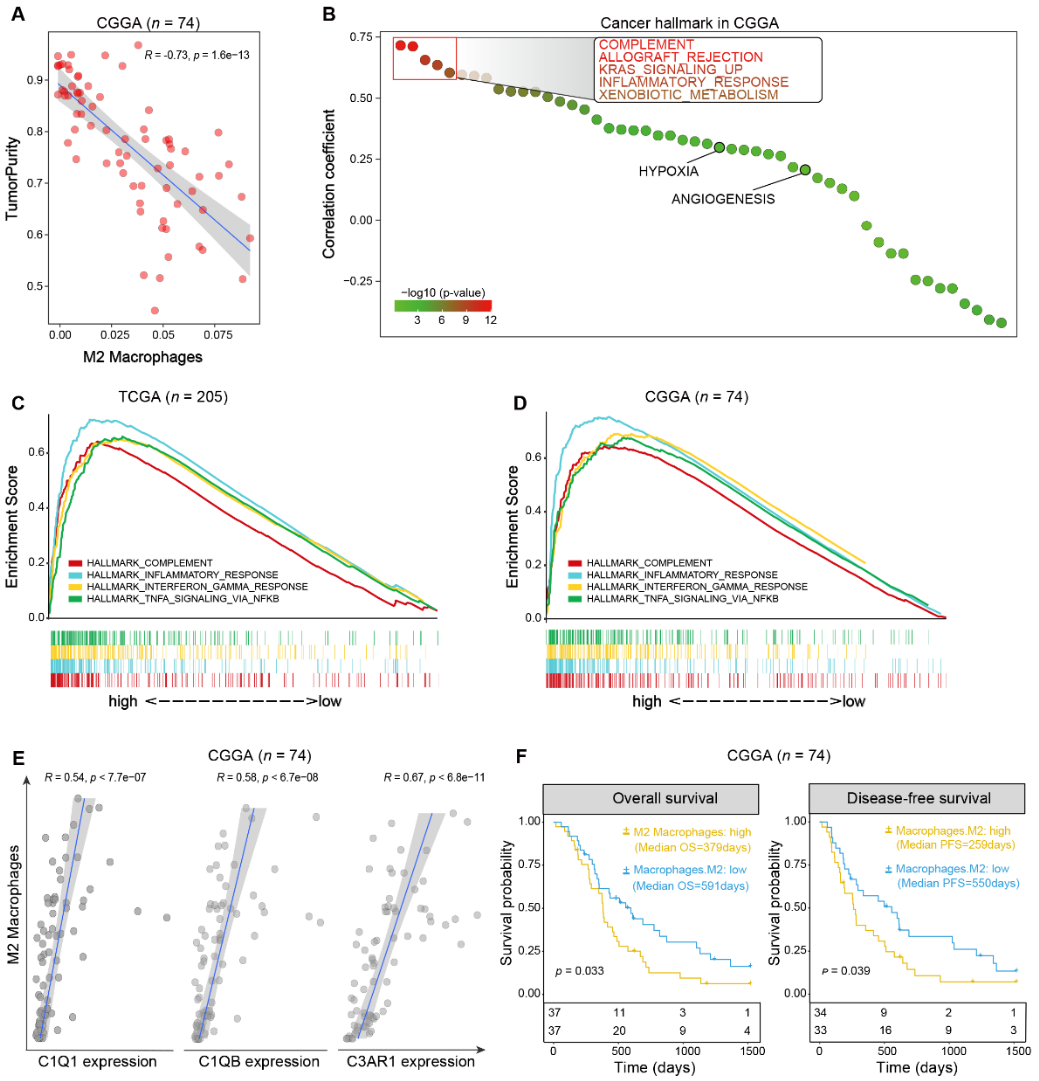

Supplementary Figure S1. High M2 macrophage infiltration is associated with reduced tumor purity, complement pathway activation, and poor prognosis in GBM in the CGGA cohort. **A,** Scatter plot showing a negative correlation between M2 macrophage scores (xCell) and tumor purity estimated by ESTIMATE in the TCGA cohort. **B,** Correlation analysis showing a positive association between M2 macrophage scores (xCell) and the Hallmark complement pathway estimated by ssGSEA. **C–D,** GSEA analysis showing enrichment of complement-related pathways in samples with high M2 macrophage infiltration. **E,** Correlation analysis showing a positive association between M2 macrophage scores (xCell) and the expression of key complement pathway genes, including C1QA, C1QB, and C3AR1. **F,** Kaplan–Meier survival curves comparing overall survival (left) and progression-free survival (right) between high and low M2 macrophage score groups in the CGGA cohort. Survival differences were assessed using the log-rank test (p values indicated).

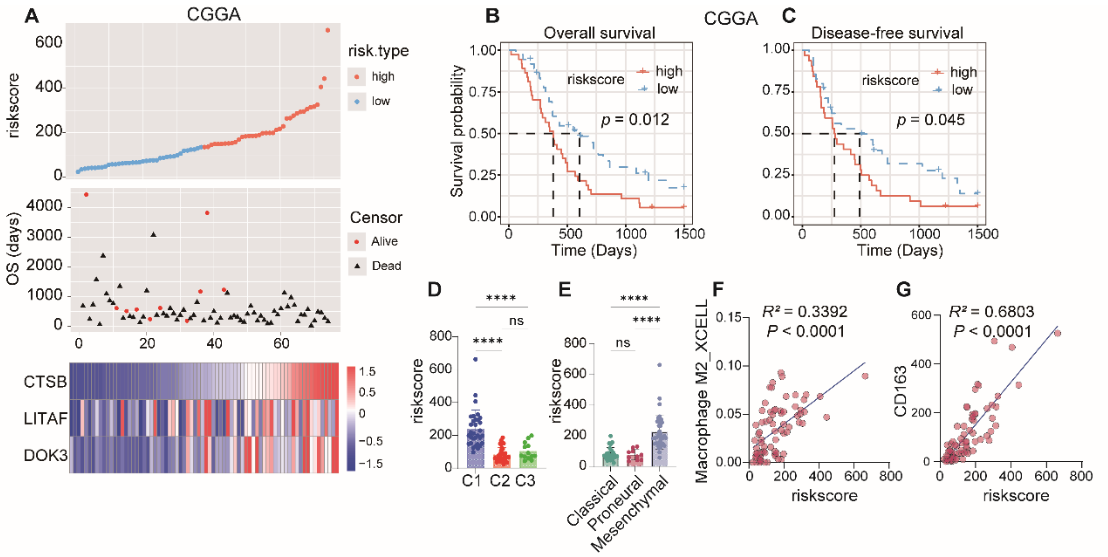

Supplementary Figure S2. Construction of a macrophage-related risk score model to identify the C1 immunosuppressive subtype in CGGA cohort. A, Using the three candidate genes to group the risk scores, it was observed that the high-risk group (orange) had a poorer prognosis compared to the low-risk group (cyan). **B-C,** Kaplan-Meier survival analysis demonstrated that higher risk scores were associated with shorter OS and PFS. **D,** Comparison of risk scores among the three immune subtypes (C1–C3). **E,** Comparison of risk scores across GBM molecular subtypes, with the highest scores observed in the mesenchymal subtype. **F-G,** Correlation of the risk score with M2 macrophage infiltration (F) and CD163 expression (G).

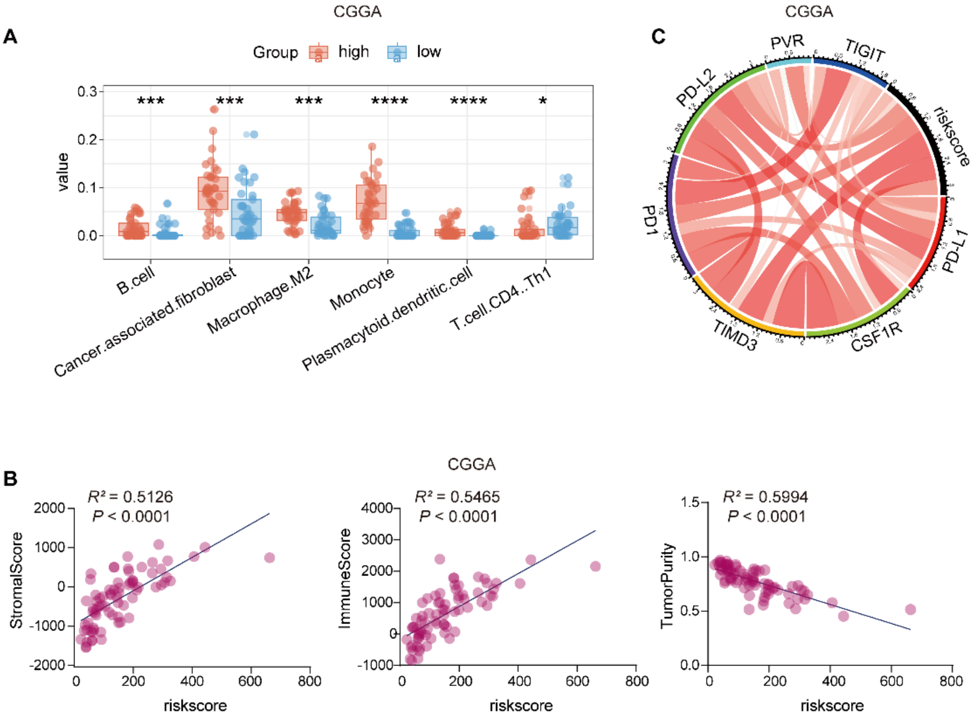

Supplementary Figure S3. Validation of the immune microenvironment features in the CGGA cohort. A, Immune cell infiltration differences between high- and low-risk groups based on xCell analysis, consistent with the CGGA cohort. **B,** Correlation of the risk score with immune score, stromal score, and tumor purity as computed by ESTIMATE. **C,** Positive correlation between risk score and the expression of immune checkpoint genes TIMD3, CSF1R, and PDCD1, confirming the immunosuppressive profile of high-risk tumors.

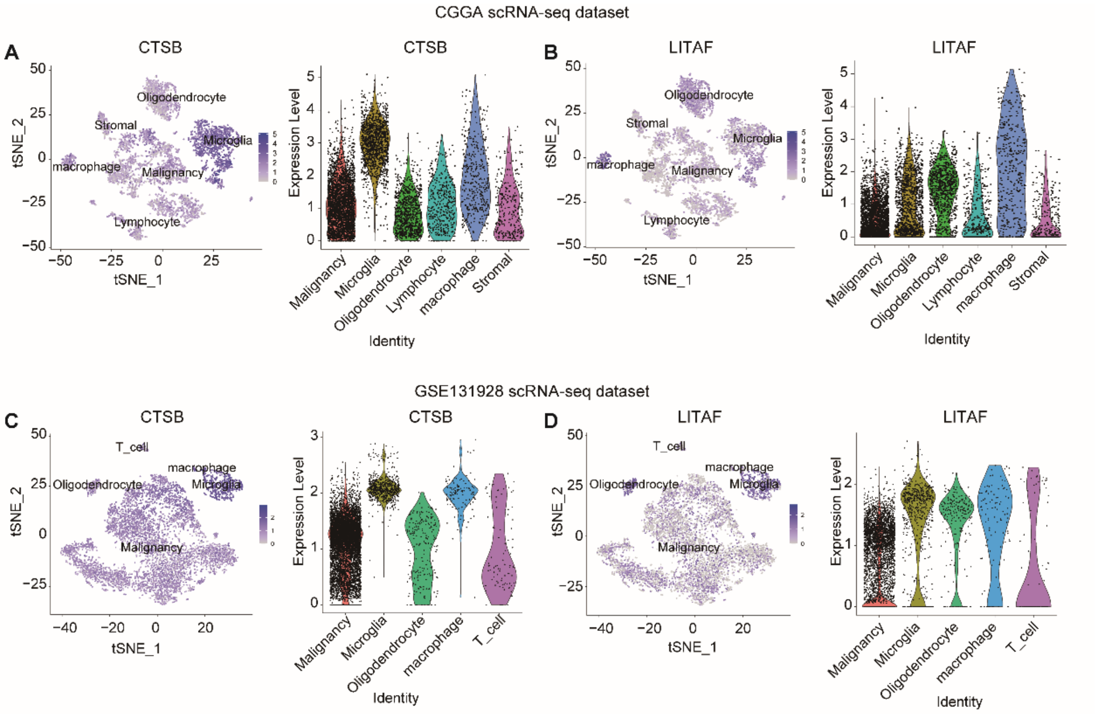

Supplementary Figure S4. CTSB and LITAF exhibit broad, non-specific expression across multiple cell types. A-D, Single-cell RNA-seq (scRNA-seq) analysis of glioma samples from the CGGA and GSE131928 datasets. UMAP plots show cell clustering and the expression of canonical myeloid markers (CD68, TMEM119, CD163) as well as CTSB (A, C) and LITAF (B, D), demonstrating that CTSB and LITAF are expressed across multiple cell types.

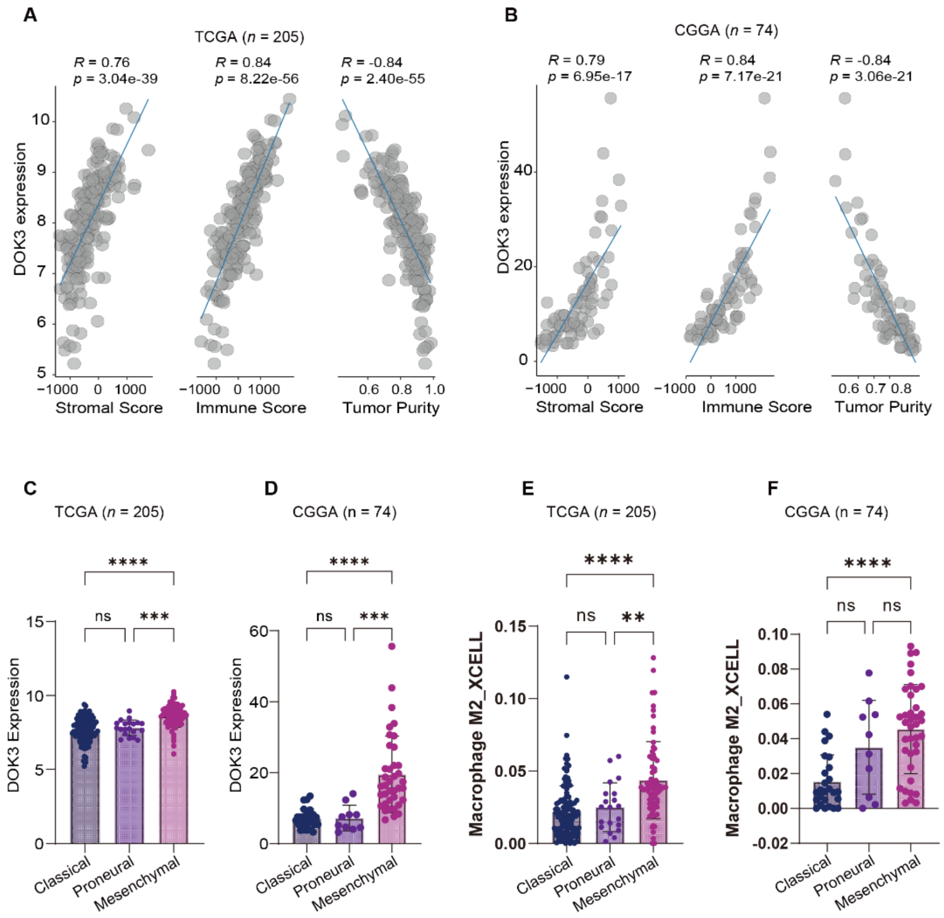

Supplementary Figure S5. Association of DOK3 expression with tumor microenvironment features and molecular subtypes. A–B, Correlation analyses showing the relationship between DOK3 expression and immune score, stromal score, and tumor purity in the TCGA (A) and CGGA (B) cohorts. DOK3 expression is positively correlated with immune and stromal scores and negatively correlated with tumor purity. **C–D,** Comparison of DOK3 expression levels across molecular subtypes, demonstrating significantly higher expression in the mesenchymal subtype compared with the classical and proneural subtypes in the TCGA (C) and CGGA (D) cohorts. **E–F,** Distribution of macrophage infiltration scores across molecular subtypes in the TCGA (E) and CGGA (F) cohorts, showing significantly increased macrophage infiltration in the mesenchymal subtype.
